# Polarization and health-related behaviors and outcomes during the COVID-19 pandemic: a systematic review

**DOI:** 10.1016/j.ssmph.2025.101891

**Published:** 2025-12-04

**Authors:** Aziz Mert Ipekci, Maximilian Filsinger, Diana Buitrago-Garcia, Cristopher I. Kobler Betancourt, Harvy Joy Liwanag, Annika Frahsa, Nicola Low

**Affiliations:** a Institute of Social and Preventive Medicine, University of Bern, Bern, Switzerland; b Multidisciplinary Center for Infectious Diseases, University of Bern, Bern, Switzerland; c Graduate School for Health Sciences, University of Bern, Bern, Switzerland; d Institute of Political Science, University of Bern, Bern, Switzerland; e ESPOL-LAB, Université Catholique de Lille, France; f NUS Saw Swee Hock School of Public Health, National University of Singapore and National University Health System, Singapore

## Abstract

**Objective:**

Political and affective polarization are different, but related concepts, which can shape trust in authorities, interpretation of health messages, and health behaviors and outcomes. The aim of this study was to systematically review the research literature, exploring how affective and political polarization are associated with COVID-19-related health behaviors and outcomes.

**Methods:**

From January 1, 2019 to November 27, 2024, we searched 12 electronic databases for studies about affective or political polarization and COVID-19-related outcomes, including preventive behaviors such as vaccination, compliance with policies, perceived risk and health outcomes. We included studies reporting primary data from participants of any age and gender, published in any language. Two independent reviewers, from a total of seven, conducted study selection, data extraction and risk of bias assessment. We synthesized findings narratively and reported them according to the PRISMA 2020 statement.

**Results:**

Of 2021 unique articles, we included nine cross-sectional studies, all conducted in the United States of America or Europe from 2020 to 2022. Four studies found associations between higher political polarization and lower COVID-19 vaccine uptake or intent. Reported associations between vaccination and affective polarization were mixed. Four studies of other COVID-19 attitudes or prevention measures found mixed results for both types of polarization. In one study, no association was found between polarization and changes in death in 2020 compared with 2015 to 2019. The risk of selection bias in included studies was high.

**Discussion:**

This systematic review found some evidence of associations between polarization and COVID-19 health-related behaviors and outcomes. Cohort studies are needed to understand the direction of association. More international and interdisciplinary approaches to the study of polarization are needed to generate evidence to inform health and public policy effectively and improve preparedness for future pandemics.

## Highlights

• Understanding of how polarization impacts COVID-19 health behaviors is fragmented.
• Higher political polarization was linked to lower COVID-19 vaccine uptake or intent.
• Associations were more consistent for political than affective polarization.
• Poor reporting of response rates and results limits assessment of risks of bias.
• Cohort studies will improve assessment of causal links with COVID-19 outcomes.

## Graphical abstract

**Figure:**
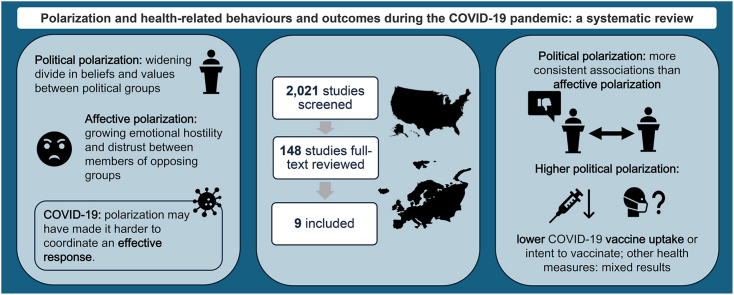

## 1 Introduction

During the COVID-19 pandemic emergency, researchers identified disparities in how people adhered to infection prevention measures, such as mask-wearing or vaccination (Kusuma et al., 2021; Patzina & Dietrich, 2022) and in health outcomes, such as infection and mortality (Sepulveda & Brooker, 2021). While researchers in health sciences often study factors such as age, gender, occupation, and socioeconomic position as determinants of health, they may overlook others, like political ideology and polarization and the emotions these ideologies evoke. Research in political sciences has shown that individuals interpret the actions of government or other authorities through ideological and partisan lenses (Bartels, 2002; Gadarian et al., 2021; Malhotra & Kuo, 2008). Given the multiple and interrelated determinants of human behaviors and health, an interdisciplinary approach to research incorporating political influences could contribute to a more comprehensive understanding of COVID-19-related health behaviors and outcomes.

Political and affective polarization are related but different concepts, which were developed by political scientists in the United States of America (USA) to understand voters’ behaviors, and are increasingly relevant to health behaviors, outcomes, and public health (Mello & Wang, 2025) (Box 1). Political polarization describes the extent of separation between political opinions and beliefs, which is often expressed as identification with political party ideology (partisanship) (Fiorina & Abrams, 2008) and also referred to as ideological polarization (Van Bavel et al., 2024). Affective polarization is also political; it captures the feelings of dislike or distrust by individuals or groups with a particular partisan identity toward those with opposing views and conversely, positive views towards those with the same identity (Iyengar et al., 2019). Political polarization can occur without affective polarization, meaning that people can hold differing political views without harboring hostile feelings toward those who disagree. Both types of polarization can be assessed quantitatively (Baldassarri & Gelman, 2008; Druckman & Levendusky, 2019) (Box 1).

Box 1 **Glossary of terms about polarization in political science**

**Ideology**

A set of beliefs, values and principles. A political ideology provides a framework for defining how society should be organized and governed, understanding political issues, interpreting political events, or deciding on preferred policies. Political ideologies are often classified along a spectrum from left to right, based on their position about issues of social equality and hierarchy. Different ideologies shape how individuals align themselves with like-minded groups, including political parties (Kteily & Brandt, 2025).

**Partisanship**

Attachment or loyalty to a political party. While political ideology is about a belief system, partisanship is about group identity. Individuals who believe strongly in a political ideology often gravitate toward the political party that best aligns with their worldview. In the United States of America, these are the Democratic and Republican parties. In multiparty systems, partisanship follows party political positions on the left-right spectrum. Partisanship can be expressed as a social identity, implying a stable identification with a political party. Partisanship is influenced by political attitudes but also affects the formation of attitudes (Wang & Klar, 2022).

**Political polarization**

The distance between political groups in terms of their policy preferences, beliefs and worldviews on the left-right political spectrum. Strong partisanship, rooted in ideological differences, amplifies polarization, as party members adopt increasing extreme political positions and move further apart from their political opponents (Fiorina & Abrams, 2008). Political polarization can be categorized at the levels of the “elite” (high-ranking members of political parties e.g., those in the parliament) and the “mass” (the general population, voters) (Fiorina & Abrams, 2008).

Political polarization at the individual level is typically measured in two steps, most often using survey questionnaires. The most common approach combines ideological distance scales with issue position scales. Large public opinion surveys, such as the American National Election Study, ask citizens to place themselves on a left–right ideological continuum and to state their preferences on a range of salient political issues. Researchers then evaluate the dispersion, bimodality, or distance between the mean positions of partisan groups in these distributions to determine the degree of polarization. Larger values on these metrics are interpreted as greater political polarization in the general public (Baldassarri & Gelman, 2008). Other methods include partisan identification strength, and perceived ideological distance (Ojer et al., 2025).

**Affective polarization**

The tendency for individuals to both disagree with members of the opposing political party and to dislike and distrust them. Affective polarization is an emotional layer of polarization and relates to the difference in feelings toward your political in-group and out-group. It is commonly understood as positive emotional attachment to in-group partisans and hostility toward out-group partisans (Iyengar et al., 2012). While political polarization describes ideological and policy gaps, affective polarization captures the negative feeling and hostility that often emerge from partisan divisions in the population (Druckman & Levendusky, 2019). Most research has focused on partisanship as the foundation of affective polarization, but it is also possible to be based on positions about specific issues (Filsinger & Freitag, 2024; Hobolt et al., 2021).

Affective polarization at the level of the individual is measured in two stages. The most common method is the feeling thermometer. First, respondents identify their position on the polarized issue (e.g., Democratic or Republican partisanship). Second, they use a numerical scale to rate how warmly or coldly they feel toward members of their own party and toward members of the opposing party. The affective polarization score is calculated as the absolute difference between these two ratings, ensuring the value is always positive. Other approaches include character trait evaluations, trust questions, and social distance measures (Druckman & Levendusky, 2019; Gidron et al., 2022; Kekkonen et al., 2022; Kubin & von Sikorski, 2021).

Alt-text: Box 1

There is growing evidence that polarization can shape health behaviors and outcomes (Van Bavel et al., 2024). In the pre-COVID-19 era, researchers in the USA investigated a range of physical and mental health outcomes and associations with political polarization. For example, individuals who perceived themselves as being more politically polarized than ‘average’ voters in their state reported more days of poor overall physical health (Fraser et al., 2022). Whilst politically polarized respondents did not report worse overall mental health, those who perceived increasing political polarization after the 2016 USA presidential election were more likely to experience new diagnoses of depressive and anxiety disorders than those who noticed no shift in polarization (Nayak et al., 2021). Krupenkin examined how political partisanship influenced childhood vaccination rates in the USA, categorizing individuals as in-partisans (those who supported the ruling government) or out-partisans (those who opposed it) (Krupenkin, 2021). Out-partisans who opposed the president at the time were less likely to follow government vaccination recommendations than in-partisans (Krupenkin, 2021). At the start of the COVID-19 pandemic, the increasing role of political ideology in health behaviors was observed (Geana et al., 2021) and Van Bavel and colleagues identified the potential for political and affective polarization to influence behavioral responses to the pandemic in opposing ways (Van Bavel et al., 2020). In 2024, Van Bavel and colleagues used the COVID-19 pandemic as a case study in a narrative review. They argued that polarization of viewpoints evolved over the course of the pandemic and undermined both individual and collective well-being (Van Bavel et al., 2024).

Polarization appears to have increased in many countries, especially during the COVID-19 pandemic, so interdisciplinary collaborations between health and social scientists should help to understand its impact on health (Van Bavel et al., 2024). Despite a growing body of research linking polarization to health outcomes, the evidence remains fragmented. Systematic reviews are an essential tool for synthesizing the findings of a body of evidence about a specific question, including polarization in relation to political participation as a social behavior (Kołczyńska, 2025; Lubej et al., 2025). The aim of this study was to systematically review the research literature, exploring how affective and political polarization relate to COVID-19-related health behaviors and outcomes.

## 2 Methods

This systematic review followed a protocol (Ipekci et al., 2024) and is registered in the online PROSPERO database (https://www.crd.york.ac.uk/PROSPERO/view/CRD42023475828). We report the review following the Preferred Reporting Items for Systematic Reviews and Meta-Analyses 2020 (PRISMA 2020) and Systematic Reviews and Meta-Analysis Synthesis without meta-analysis guidelines (Campbell et al., 2020; Page et al., 2021) (Appendix A. Supplementary material 1). We used Covidence systematic review software to screen records and extract data from included studies (Veritas Health Innovation, Melbourne, Australia, www.covidence.org).

### 2.1 Search strategy

We developed the search strategy with an information specialist using predefined terms for polarization and COVID-19 (Supplementary material 3). We searched 12 electronic databases from January 1, 2019, initially until September 8, 2023 and again until November 27, 2024, covering different research fields and publication types: EMBASE, Medline (Ovid), Cochrane Library, Cochrane COVID-19 Study Register, Global Health (Ovid), PsycInfo (Ovid), Web of Science, CINAHL, EconLit (EBSCOhost). We searched the World Health Organization (WHO) COVID-19 Database and iSearch COVID-19 Portfolio (USA National Institutes of Health) for preprints. To identify additional studies, we searched Google Scholar, reviewed the reference lists of relevant studies and systematic reviews and contacted experts in the field. We used reference management software (EndNote – Clarivate, version 20.4, www.endnote.com) to manage the retrieved records.

### 2.2 Eligibility criteria

Studies were eligible for inclusion if they included primary data from individuals of any age and gender and assessed affective or political polarization or both quantitatively as the exposure, irrespective of publication status or language (Supplementary material 4). We included studies where definitions of political and affective polarization were in line with those used in political science (Box 1). Eligible outcomes comprised COVID-19 infection risk, hospitalization, mortality, vaccine uptake, compliance with measures such as mask-wearing and physical distancing, and perceived COVID-19 risk. Eligible study designs were cohort studies, case-control studies, cross-sectional studies, and ecological studies. We excluded studies that only analyzed data from social media platforms for two methodological reasons. First, in social media-based studies, measures of polarization focus on sentiments within user networks, which often do not capture the main features of affective polarization, as defined in political science (Hohmann & Coscia, 2025). Second, the way that the measures are derived for example from text analyses or network analyses of Twitter/X posts (Hohmann et al., 2023), cannot be compared with self-report or aggregated responses from surveys or elections.

### 2.3 Study selection

Two reviewers screened all titles and abstracts independently and selected potentially relevant articles, according to the eligibility criteria. Disagreements were resolved through discussion or by a third reviewer. Two independent reviewers retrieved the full text of all potentially eligible articles independently. In case of disagreements that were not resolved by discussion, a third reviewer decided.

### 2.4 Data extraction

We developed, piloted, and revised a data extraction form. Two independent reviewers extracted data from each study. Disagreements were resolved through discussion or by a third reviewer. We extracted data on: study level characteristics, measurement of polarization and COVID-19-related health behaviors or outcomes, the main findings, and possible confounding factors (Supplementary material 5).

### 2.5 Dealing with missing data

We contacted corresponding authors in case of any missing data. If the author did not reply, two reviewers decided whether the study could still be included.

### 2.6 Quality and risk of bias assessment

Two independent reviewers assessed the quality of included studies using the JBI checklist for analytical cross-sectional studies, which primarily focuses on reporting quality (Joanna Briggs Institute, 2017). We added five items about risk of bias, using a tool published in a systematic review about COVID-19 (Tonia et al., 2023). Disagreements were resolved through discussion or by a third reviewer.

### 2.7 Data synthesis and analysis

We employed narrative synthesis methods (Campbell et al., 2020), starting with a description of study characteristics, methods, and participant characteristics. We grouped outcomes as: (1) vaccine uptake (reported receipt or completion of vaccination) or vaccine intent (expression of willingness or plan to get vaccinated), (2) compliance with COVID-19 prevention measures, and (3) “other outcomes” (worry about COVID-19 and COVID-19 deaths). We summarized study findings in a table and in the text. We visualized the study findings and characteristics in a harvest plot (Ogilvie et al., 2008).

### 2.8 Research team

The team of seven reviewers included epidemiologists, political scientists, anthropologists, health policy experts, systematic review methodologists and an information scientist, with a range of language and cultural backgrounds.

### 2.9 Differences between published protocol and the manuscript

Four deviations from the protocol occurred: (1) two independent reviewers screened all studies because the Covidence tool does not support the planned approach for one reviewer to screen and one to verify decisions; (2) to ensure conceptual consistency across studies, we refined our inclusion criteria to require definitions of political and affective polarization that were in accordance with political science literature; (3) we excluded studies based solely on social media data because their definitions and measures are too different from survey-based studies; and (4) we included one cross-sectional study in which affective polarization was specified as the outcome, rather than the exposure because the interpretation still addressed our review question. Full details are provided in Supplementary Material 2.

## 3 Results

### 3.1 Search results

**Fig. 1:**
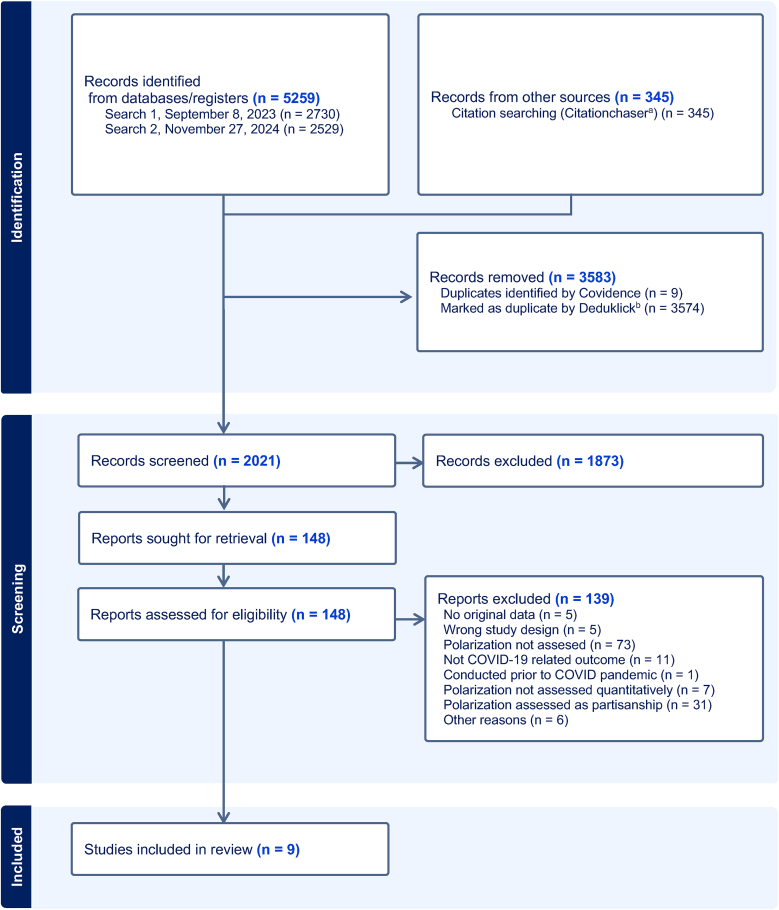
Flowchart for included studies. *Notes:* a. Citationchaser is a Shiny app in RStudio for forward and backward citation chasing in academic searching (https://estech.shinyapps.io/citationchaser/); b. Deduklick is a software application, which identifies and removes duplicate records from bibliographic datasets (https://www.risklick.ch/deduklick).

Our search yielded a total of 5259 studies. After removal of duplicates, 2021 studies were screened (Fig. 1). Of these, 1873 were excluded during title and abstract screening. Of 148 studies assessed as full text, we included nine. Of these, eight met the inclusion criteria, with affective or political polarization or both specified as the exposure (Charron et al., 2023; Cornelson & Miloucheva, 2022; Dolman et al., 2023; Druckman et al., 2021; Hierro et al., 2023; Kim & Pelc, 2024; Wagner & Eberl, 2024; Wróblewski & Meler, 2024). We included one cross-sectional study, in which affective polarization was specified as the outcome (Nezi, 2024). In that study, the exposure and the outcome were measured in the same survey. The direction of association between the two variables could therefore not be determined, so the interpretation of the findings fulfilled the criteria for our review. The reasons for exclusion of studies are summarized in the flow chart. The main reasons for exclusion were that the study did not assess polarization (n = 73), the study measured political identity (partisanship) and not polarization (n = 31), e.g., (Block et al., 2022; Gao & Radford, 2021), or the study used only social media data, e.g., (Ebeling et al., 2022). The list of studies that were excluded after reading the full text and the reasons are provided in Supplementary material 6.

### 3.2 Characteristics of included studies

The data for the nine included studies were all collected while the WHO still considered COVID-19 a public health emergency of international concern (World Health Organization, 2023) (Table 1). Five studies were conducted during 2020, before vaccines were available (Charron et al., 2023; Cornelson & Miloucheva, 2022; Druckman et al., 2021; Hierro et al., 2023; Wróblewski & Meler, 2024), two during 2021 (Dolman et al., 2023; Kim & Pelc, 2024) and two during 2022 (Nezi, 2024; Wagner & Eberl, 2024). Four studies were conducted in the USA (Cornelson & Miloucheva, 2022; Dolman et al., 2023; Druckman et al., 2021; Kim & Pelc, 2024), one in Austria (Wagner & Eberl, 2024), one in Greece (Nezi, 2024) and three in multiple member states of the European Union (Charron et al., 2023; Hierro et al., 2023; Wróblewski & Meler, 2024). We did not find any eligible studies conducted in Africa, Asia, Latin America, or Oceania. In six of nine included studies, all authors were from the same discipline (Cornelson & Miloucheva, 2022; Druckman et al., 2021; Hierro et al., 2023; Kim & Pelc, 2024; Nezi, 2024; Wróblewski & Meler, 2024). Among 27 authors of the articles, there were 30 affiliations, of which 14 were departments of political science; eight economics; four social science; two health science and one communications. Only two authors listed an affiliation in a department of health sciences (Dolman et al., 2023).

**Table 1:** Characteristics of nine included cross-sectional studies, alphabetical order.

First author (publication year); data collection years^a^	Country	Exposure; data type;^b^ measurement method	Polarized groups (authors' description)	Outcome; data type;^b^ measurement method	Number of participants analyzed	Main results and interpretation
a. Charron et al. (2023); 2020	165 regions in 20 European countries	Affective and political polarization; individual and ecological; survey	Partisanship-related polarization; government supporters vs. non-supporters (Difference in trust)	Percentage increase or decrease in death in 2020 compared with 2015–19; ecological; data from national statistical office	Not reported	Affective polarization: No strong statistical evidence of association between trust gap (supporters vs. non-supporters) in a region and percentage change in deaths in 2020 vs. 2015-19 in a region Coefficient 1.49 (95 % CI, −2.84, 5.81) Political polarization: Some evidence of association between polarization among political parties in a region and percentage change in deaths in 2020 vs. 2015-19 in a region, controlling for COVID-19 cases Coefficient 2.74 (95 % CI −0.10, 5.57)
b. Cornelson and Miloucheva (2022); 2020	24 states in USA	Affective polarization; individual; feeling thermometer (scored 1–100)	Partisanship-related polarization (Republicans vs. Democrats)	Compliance with the number of COVID-19 control measures (0–9);^c^ individual; survey	1753	Those in opposing party to state's governor and polarized reported being more likely to follow more preventive behaviors than those in opposing party to state's governor and not polarized Coefficient 0.23 (95 % CI 0.03, 0.44)
c. Dolman et al. (2023); 2021^a^	USA	Political polarization; individual, survey; perceived polarization scale 0-10	Partisanship-related polarization (Republicans vs. Democrats)	Vaccination uptake and intent to take vaccine; individual; survey	1427	Low polarization group: some evidence of lower reported vaccination intent Republicans vs. Democrats OR 0.59 (95 % CI 0.34, 1.10) High polarization group: lower reported vaccination intent Republicans vs. Democrats OR 0.10 (95 % CI 0.05, 0.19) Similar patterns for vaccination uptake but smaller effect sizes (no CIs reported)
d. Druckman et al. (2021); 2019–2020	USA	Affective polarization; individual; feeling thermometer/social distance measure/trust questions/character trait	Partisanship-related polarization (Republicans vs. Democrats)	Worry about COVID-19, compliance with COVID-19 control measures^d^ (0–14), support for COVID-19 policies; individual; survey	2064	Polarized Republicans reported less worry about COVID-19 as polarization increased Coefficient −0.33 (95 % CI -0.10, −0.55) No associations between affective polarization and changes in behavior Coefficient 1.80 (95 % CI -0.98, 4.58) Polarized Republicans reported less support for COVID-19 policy as polarization increased Coefficient −0.44 (95 % CI -0.12, −0.75)
e. Hierro et al. (2023); 2020	26 European Union member states included	Affective and political polarization (combined measure); ecological; polarization index	Partisanship-related polarization	Vaccination uptake at peak of uptake; ecological; publicly available website	Not reported	One unit increase in polarization index (0–10) associated with lower vaccination uptake Coefficient −6.77 (95 % CI -0.05, −13.48)
f. Kim and Pelc (2024); 2021^a^	USA	Affective and political polarization; individual; feeling thermometer/survey	Partisanship-related polarization (Republicans vs. Democrats)	Vaccination uptake; ecological; cooperative election study	23,908	Increase in distance state's average ideology (1–5) associated with decrease in vaccination uptake Coefficient −0.02 (95 % CI -0.03, −0.02) No strong statistical evidence of association between affective polarization and vaccination uptake Coefficient 0.49 (95 % CI −1.91, 2.88)
g. Nezi (2024); 2022^a^	Greece	Level of agreement with the COVID-19 control measures, including vaccination (1–3); individual; survey	Partisanship-related polarization (In-party vs. Out-party)^e^	Affective polarization (0–10); individual; feeling thermometer/Wagner index (Wagner, 2021) (spread of like-dislike scores)	844	Increase in citizens in agreement (1–3) with mandatory vaccination associated with decrease in affective polarization Coefficient −0.26 (95 % CI -0.50, −0.03)
h. Wagner and Eberl (2024); 2020–2022∗	Austria	Affective polarization; individual; character trait measure	Vaccine supporters vs. vaccine opposers	Level of agreement with COVID-19 control measures; individual; survey	810	Increase in affective polarization (vaccine supporters vs. opposers) (−1 to 1) associated with increased support to restrict unvaccinated individuals (−1 to 1) Coefficient 0. 23 (95 % CI 0.16, 0.30)
i. Wróblewski and Meler (2024); 2020	23 European countries	Political polarization; individual; survey, polarization index	Partisanship-related polarization (In-party vs. Out-party)^e^	Vaccine uptake; individual; survey	Not reported	Increase in polarization index associated with decreased vaccine uptake by 8 % (no CIs reported)

Abbreviations: CI, confidence interval; OR, odds ratio.

a Indicates data collected after 2021, following the start of COVID-19 vaccine roll-out in the country in which the study was done.

b Individual-level or ecological-level data. Ecological-level data refers to aggregated measures that summarize information for groups or populations rather than for individual participants.

c Control measures: Non-essential travel, work outside of home, work outside home by choice, wash hands, stay home, cancel travel, limit contact, wear personal protective equipment, other.

d Behaviors: washed your hands more frequently, worked from home, used hand sanitizer, cancelled planned travel, avoided gatherings of more than ten people, tried to stay at least six feet away from other people, worn a face mask, worn gloves, did not go to a grocery store to avoid contact with others, ordered grocery delivery to avoid going to the grocery store, cooked at home to avoid ordering food handled by others, went outside less frequently to avoid contact with others, stayed at home entirely, bought extra food.

e In-party: the political party that currently holds executive power (e.g., the presidency or government). Out-party: the political party that is not in power and serves as the opposition.

**Fig. 2:**
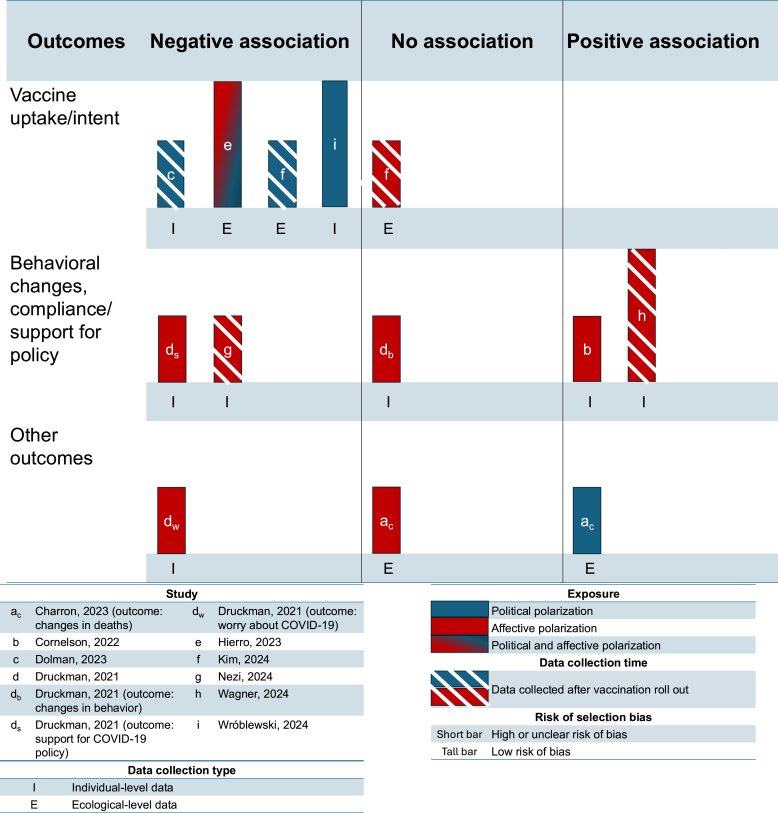
Harvest plots summarizing the association between affective and/or polarization and COVID-19-related health outcomes/behaviors. *Notes:* Study identifier: letters a – i, correspond to the letters and alphabetical order of first authors in Table 1. Exposure: red bars, affective polarization; blue bars, political polarization; red and blue bars, both types. Data collection time: diagonal stripes, data collected after COVID-19 vaccine rollout (post-2021); plain bars, data collected before vaccine rollout (pre-2021). Data collection type: I, individual-level data collection for both exposure and outcome; E, ecological-level data collection for exposure, outcome, or both. Risk of selection bias: full height, low risk of selection bias; reduced height, high or unclear risk of selection bias.

All studies used survey data in cross-sectional designs (Table 1). In eight studies, authors used individual-level data, in three, they analyzed ecological data aggregated at state or national level. Charron et al. and Hierro et al. analyzed ecological-level data for both the exposure and the outcome (Charron et al., 2023; Hierro et al., 2023), whereas Kim and Pelc included ecological-level data only for the outcome (Kim & Pelc, 2024) (Table 1; Fig. 2).

The total number of individuals invited to participate was not reported in any study. In the six studies reporting sample size, the number of included participants ranged from 810 to 23,908 (median 1590, interquartile range 1220). Details about participants’ age, gender, and socioeconomic status were frequently missing or inconsistently reported. Age and sex or gender were reported in only two studies (Dolman et al., 2023; Druckman et al., 2021). The reported ages ranged from 18 to 65 and older. No authors reported how data on sex or gender were collected and this was reported as a binary characteristic, with about half of participants reported as women. Two studies included information about education and income levels (Dolman et al., 2023; Druckman et al., 2021).

### 3.3 Risk of bias

Five studies clearly reported their inclusion and exclusion criteria, while four studies provided insufficient information (Supplementary material 7). Four studies provided detailed information about study subjects and settings, four did not, and one was judged as unclear. Eight studies included information about confounding factors, and seven studies employed appropriate methods to control for them. All studies reported the statistical analyses employed.

We identified major issues related to selection bias (Supplementary material 7). Six studies were assessed as having either a high or unclear risk based on the sampling strategies for participant inclusion. Only one study provided information on non-responders; the other eight studies were judged as unclear because this information was not reported. None of the included studies reported details about respondents and non-respondents. For exposure measurement, seven studies were judged as low risk of bias. We judged two studies as having an unclear risk of measurement bias because the polarization index developed by the authors did not allow clear assessment of associations between polarization and the outcomes (Hierro et al., 2023; Wróblewski & Meler, 2024). The measurement of outcomes was judged as low risk of bias in all studies.

### 3.4 Polarization and COVID-19-related outcomes

Two studies measured political polarization, four measured affective polarization, and three included measures of both (Table 1, Fig. 2). Eight studies examined polarization based on partisanship, that is, divisions aligned with political party affiliation (Charron et al., 2023; Cornelson & Miloucheva, 2022; Dolman et al., 2023; Druckman et al., 2021; Hierro et al., 2023; Kim & Pelc, 2024; Nezi, 2024; Wróblewski & Meler, 2024). One study defined polarization as related to vaccine attitudes, distinguishing groups based on their views toward vaccination rather than political identity (Wagner & Eberl, 2024). Polarization was most commonly measured using feeling thermometer scales but other approaches, such as social trait assessments, and character trait evaluations were also used (Table 1).

Four studies examined COVID-19 vaccine uptake or vaccine intent as the outcome, two in the USA (Dolman et al., 2023; Kim & Pelc, 2024) and two in Europe (Hierro et al., 2023; Wróblewski & Meler, 2024) (Table 1). In all studies, polarized groups were defined according to partisanship-related polarization and measured political polarization as an outcome; two studies also measured affective polarization (Hierro et al., 2023; Kim & Pelc, 2024). At least some findings in all studies suggested an inverse association; as political polarization increased, vaccine uptake or vaccine intent decreased. Dolman et al. found some evidence that, at the individual level, Republicans with low levels of polarization were less likely than their Democratic counterparts to report COVID-19 vaccine intent. In this study, highly polarized Republicans had even lower odds of intending to vaccinate than highly polarized Democrats. However, the authors found no strong evidence of association between affective polarization and vaccine uptake (Dolman et al., 2023). Kim and Pelc found that, among individuals in the USA, an increase in a person's ideological distance from their state's average (measured on a 1–5 scale) was associated with a decrease in the likelihood of being vaccinated. They also examined affective polarization and found no association with vaccine uptake (Kim & Pelc, 2024). Both studies conducted in Europe examined estimates of COVID-19 vaccine uptake across European Union member states using a polarization index (Hierro et al., 2023; Wróblewski & Meler, 2024). In both studies, an increase in the polarization index was associated with a decrease in vaccine uptake (Table 1). Two studies were conducted before COVID-19 vaccine roll-out (Hierro et al., 2023; Wróblewski & Meler, 2024) and two were conducted after (Dolman et al., 2023; Kim & Pelc, 2024). There was no clear pattern of associations according to vaccine roll-out date (Fig. 2). Two other studies, which considered other aspects of COVID-19 vaccination, are described below.

Study characteristics and findings were more mixed for four studies examining compliance with, or support for, a range of COVID-19 prevention measures or outcomes (Table 1; Fig. 2) (Cornelson & Miloucheva, 2022; Druckman et al., 2021; Nezi, 2024; Wagner & Eberl, 2024). Two studies in the USA examined associations between partisanship-related affective polarization during the first COVID-19 wave in early 2020, when a range of COVID-19 preventive measures was recommended. Cornelson and colleagues studied workers using an online survey. They found that participants who were in the opposing party to their state's governor and categorized as affectively polarized followed more preventive behaviors (such as staying at home and limiting in-person contact with high-risk individuals) than those in opposing states and not polarized (Cornelson & Miloucheva, 2022). Druckman et al. studied a nationally representative sample to investigate the association between affective polarization and attitudes or behaviors related to COVID-19. They found that affectively polarized Republicans were less likely to worry about COVID-19 as polarization increased and less likely to support COVID-19 policies as affective polarization increased but found no association between affective polarization and behaviors (Druckman et al., 2021). The other two studies were conducted in Europe. Nezi conducted a study in Greece, which defined the exposure as support for a policy of mandatory vaccination and partisanship-related affective polarization as the outcome. Among all participants, an increase in support for vaccination was associated with a decrease in affective polarization (Nezi, 2024). Wagner and Eberl measured affective polarization in relation to support or opposition to COVID-19 vaccination in Austria. In a series of individual-level online surveys between 2020 and 2022, they found that an increase in affective polarization was associated with an increase in support for restriction of unvaccinated individuals (Wagner & Eberl, 2024). There was only one study with a direct health outcome. Charron and colleagues studied mortality as an outcome, at the national level in European Union member states. They found no association between affective polarization and percentage increase or decrease in death in 2020 compared with 2015–19. However, they found some evidence of a relationship between political polarization and change in death rates over time (Charron et al., 2023).

## 4 Discussion

### 4.1 Summary of main results

This systematic review found that associations between political polarization and health outcomes were more consistent than those for affective polarization. In four studies, higher levels of political polarization were associated with lower vaccine uptake or vaccine intent. Studies about compliance or support for COVID-19 prevention measures were more mixed. Two studies found that polarized individuals were more likely to adopt preventive behaviors or support stricter measures (such as vaccine mandates or restrictions on the unvaccinated); two others reported negative or null associations between polarization and these outcomes. All included studies were cross-sectional in design, so conclusions about the direction of association cannot be made. No studies were conducted in countries other than the USA and member states of the European Union.

### 4.2 Strengths and weaknesses of the systematic review methods

Strengths of our study include our multidisciplinary review team, which involved experts in epidemiology, social sciences, and political science at all stages of conceptualization, conduct and interpretation of the findings. In contrast, the authors of the individual included articles were usually affiliated with a single discipline, with few from health science departments. We registered and published our protocol (Ipekci et al., 2024), defining our methods in advance and following reporting guidelines, to aid reproducibility.

There were limitations and challenges for the systematic review method, as applied to our multidisciplinary review question. We studied concepts of polarization from the political sciences and health-related behaviors and outcomes, defined in epidemiological terms. Systematic reviews were developed to summarize quantitative data and assess risks of bias in randomized controlled trials of healthcare interventions, and were then adapted to other study designs (Page et al., 2021). Our protocol aligned with the norms and structure of reporting of observational epidemiological studies (Von Elm et al., 2007), which are also followed by systematic reviews in healthcare. The studies included in our review were all led by researchers in the social sciences, including political and economic sciences. In these disciplines, the narrative style of describing methods and results follows a structure different from health sciences, which resulted in challenges to both data extraction and synthesis of findings. For instance, none of the studies reported the numbers of people invited to participate or compared responders and non-responders, which are essential items for assessing risks of selection bias. Our study had additional limitations. First, despite a broad search strategy, we did not search grey literature sources, so we might have missed unpublished studies. We did, however, search preprint servers. Second, while meta-analysis would have been useful to quantify summary measures of association, this was not possible. Rather, a narrative approach was appropriate, given the limited number of included studies and differences between them in the COVID-19-related outcomes, the type of polarization studied and the tools used to measure and quantify it, and in the statistical presentation of the results.

### 4.3 Comparison with other systematic reviews on polarization

Van Bavel and colleagues have published a narrative review of political polarization and health (Van Bavel et al., 2024), but to our knowledge, no other systematic review has examined the relationship between polarization and health behaviors and outcomes, including those related to COVID-19. We found two systematic reviews about polarization and political participation as a social behavior, which provide useful points of comparison for our study. Using system-level polarization as the reference group, a systematic review and meta-analysis of 25 studies found a strong positive relationship between affective (coefficient 0.04, 95 % CI 0.04, 0.05) and political (coefficient 0.03, 95 % CI 0.01, 0.06) polarization and political participation. However, the authors cautioned that publication bias (Thornton & Lee, 2000) favoring positive results may have influenced the conclusions (Kołczyńska, 2025). Lubej and colleagues included 18 studies in a systematic review of affective polarization and political participation and reached the same conclusion in their narrative synthesis. The authors noted the challenge in synthesizing data from studies that use different methods to measure affective polarization (Lubej et al., 2025). Both reviews had larger numbers of studies than ours, including studies from Asia and Latin America, which might indicate that interest in polarization and health is more recent, or simply that the COVID-19 pandemic only began in 2020.

### 4.4 Interpretation of the review findings

Overall, we found some evidence of association between both political and affective polarization and COVID-19 health behaviors and outcomes, suggesting a divide in health behaviors among polarized individuals. The relationships between both forms of polarization and health behaviors and outcomes are often conditional on political group membership, such as partisanship. Polarization does not have a uniform effect on health behaviors and attitudes; rather, its influence depends on the individual's in-group affiliation. For example, Druckman et al. found that affective polarization was negatively related to support for COVID-19 policies among Republicans, but not among Democrats (Druckman et al., 2021).

We identified five specific factors, which should be considered in interpretation of our findings. First, the review included only nine studies, which makes it difficult to determine whether differences in findings are due to chance or varying methodological approaches. Several studies examined multiple exposure-outcome associations and subgroups; therefore biases related to selective reporting and preferential publication of “statistically significant” results might have influenced the findings we report (Thornton & Lee, 2000). Second, most studies were either at high risk of selection bias, because of the methods used to include participants, or unclear risk because the proportion of participants taking part was not reported (Supplementary material 7). Since no study reported a comparison of respondents and non-respondents, the presence or absence of bias was unknown. It is therefore not possible to say whether observed associations were likely to be over- or under-estimated. Third, all studies included in our review were cross-sectional, so the direction of association is unknown and causal inference is not possible. Fourth, in included ecological studies, e.g. (Charron et al., 2023; Hierro et al., 2023; Kim & Pelc, 2024), associations at group level do not necessarily apply at the individual level. Fifth, two studies did not consistently report confidence intervals (Dolman et al., 2023; Wróblewski & Meler, 2024). Whilst this absence limits interpretation of statistical uncertainty, we do not believe our conclusions are affected.

Associations between political polarization and health outcomes were more consistent than those for affective polarization. In particular, we found some evidence of an inverse association between increasing levels of political polarization and decreasing levels of COVID-19 vaccine uptake or vaccine intent. This finding reflects, in part, that these were the most commonly measured exposure and outcome. The association was seen both in studies conducted in the USA (Dolman et al., 2023; Kim & Pelc, 2024) and Europe (Hierro et al., 2023; Wróblewski & Meler, 2024), which suggests that the measures of partisanship from the two-party USA political system were adapted appropriately to multiparty systems, which are common in Europe.

The relationship between both political and affective polarization and the broader range of COVID-19 health behaviors was more variable than for vaccination for two main reasons. First, vaccination-related behavior is easier to define and measure in ways that can be compared between studies. COVID-19 vaccination emerged as a central point of political conflict during the pandemic, perhaps making it more likely to be studied than other issues, and to reveal underlying patterns of polarization (Filsinger & Freitag, 2024, 2025). Second, the range of preventive behaviors investigated was wide, with up to 14 different measures (Druckman et al., 2021), and different measures of polarization, which made it difficult to draw out common patterns.

### 4.5 Implications for research and practice

Understanding the factors that influence health outcomes and behaviors during a pandemic, such as COVID-19, should make an essential contribution to improving preparedness and response strategies for future pandemics. Our review suggests relationships between affective and political polarization and COVID-19-related behaviors and health outcomes, but also reveals gaps, for which further research is needed. First, all included studies were conducted in the USA or Europe and by political or economic scientists, which limits generalizability to regions where political, cultural, economic, and health system factors differ. Studies using public-health-led designs from other countries and settings are therefore needed to broaden understanding of the relationship between political and affective polarization and health in diverse sociocultural contexts. Second, COVID-19 vaccination uptake or vaccine intent are proxies for direct health outcomes; we found only one study with mortality as the outcome (Charron et al., 2023). Future studies should explore how polarization influences health outcomes, including infection rates, and mortality, to clarify the broader public health implications. Furthermore, whilst we defined polarization as the exposure, whereby it might affect a person's health-related behaviors and outcomes, researchers should tackle the question of whether preventive measures against pandemics may be the source of polarization themselves, as suggested by recent studies (Filsinger & Freitag, 2024, 2025; Wagner & Eberl, 2024). Cohort studies or panel surveys, with repeated polarization and health measures are needed to determine temporality. To understand causality, multivariable analyses in observational studies, and experimental designs, including natural experiments that exploit policy discontinuities, will help to reduce confounding. To ensure public health relevance, analyses should explore potential effect modification by social and societal factors, and institutional trust. For all study designs, preregistration of protocols, with clear definitions and prespecified plans for analysis of both exposure and outcome are needed.

The topic of health-related behavior in the context of political polarization inherently spans multiple disciplines, including health sciences, political science, and social sciences. There is a broader issue about the need for understanding and explanation by researchers from different disciplines, when examining a shared topic (Van Baalen & Boon, 2024). More interdisciplinary approaches would move beyond perspectives from a single discipline, where researchers operate within the boundaries of their own fields. To improve the value of primary studies and systematic reviews in this field, the principles of epidemiological research reporting and synthesis are applicable to quantitative social sciences. Standardization of definitions and measurement of polarization, and adherence to standards for transparent and complete reporting of methods and results would be beneficial. Interdisciplinary research is essential for addressing complex questions, like understanding how political polarization affects health, as authoritarian government exerts increasing political control over health research and policy (The Lancet, 2025). Overall, our systematic review findings about the associations between polarization and COVID-19-related health outcomes underscore the potential to generate the evidence to inform health and public policy effectively, and improve preparedness for future pandemics.

## CRediT authorship contribution statement

**Aziz Mert Ipekci:** Writing – review & editing, Writing – original draft, Methodology, Data curation, Conceptualization. **Maximilian Filsinger:** Writing – review & editing, Data curation, Conceptualization. **Diana Buitrago-Garcia:** Writing – review & editing, Methodology, Data curation. **Cristopher I. Kobler Betancourt:** Writing – review & editing, Data curation. **Harvy Joy Liwanag:** Writing – review & editing, Data curation. **Annika Frahsa:** Writing – review & editing, Supervision, Funding acquisition, Data curation. **Nicola Low:** Writing – review & editing, Writing – original draft, Supervision, Methodology, Conceptualization.

## Ethics and consent

Ethical approval and written consent were not required.

## Declaration of generative AI and AI-assisted technologies in the writing process

During the preparation of this work the author(s) used [Grammarly] in order to check/correct for grammatical errors. After using this tool/service, the author(s) reviewed and edited the content as needed and take(s) full responsibility for the content of the publication.

## Funding

This study was funded by the Multidisciplinary Center for Infectious Diseases, University of Bern.

## Competing interests

No competing interests were disclosed.

## Data Availability

No primary data were used for the research described in the article.
