# Supplementary material for: Polarization and health-related behaviors and outcomes during the COVID-19 pandemic: a systematic review
Source: SSM Popul Health. 2025 Dec 4;33:101891. doi: 10.1016/j.ssmph.2025.101891 (PMC12765248; doi:10.1016/j.ssmph.2025.101891)
Supplement: Multimedia component 1 [file mmc1.docx]

# Appendix A. Supplementary material

Contents

[Appendix A. Supplementary material 1](#_Toc206489059)

[Supplementary material 1a: Preferred Reporting Items for Systematic reviews and Meta-Analyses 2020 (PRISMA 2020) 2](#_Toc206489060)

[Supplementary material 1b: Synthesis Without Meta-analysis (SWiM) reporting items 6](#_Toc206489061)

[Supplementary material 2: Differences between the published protocol and manuscript 8](#_Toc206489062)

[Supplementary material 3: Search strategy 9](#_Toc206489063)

[Supplementary material 4: Eligibility criteria 14](#_Toc206489064)

[Supplementary material 5: Data extraction form 15](#_Toc206489065)

[Supplementary material 6: Studies that were excluded at full-text reading and reasons 18](#_Toc206489066)

[Supplementary material 7: Risk of bias assessment 35](#_Toc206489067)

# Supplementary material 1a: Preferred Reporting Items for Systematic reviews and Meta-Analyses 2020 (PRISMA 2020)

| Section and Topic | Item # | Checklist item | Location where item is reported* |
| --- | --- | --- | --- |
| TITLE | | |  |
| Title | 1 | Identify the report as a systematic review. | Title |
| ABSTRACT | | |  |
| Abstract | 2 | See the PRISMA 2020 for Abstracts checklist. | Abstract |
| INTRODUCTION | | |  |
| Rationale | 3 | Describe the rationale for the review in the context of existing knowledge. | Introduction p 1-4 |
| Objectives | 4 | Provide an explicit statement of the objective(s) or question(s) the review addresses. | Introduction p 4 |
| METHODS | | |  |
| Eligibility criteria | 5 | Specify the inclusion and exclusion criteria for the review and how studies were grouped for the syntheses. | Methods p 3 |
| Information sources | 6 | Specify all databases, registers, websites, organisations, reference lists and other sources searched or consulted to identify studies. Specify the date when each source was last searched or consulted. | Methods p 2 |
| Search strategy | 7 | Present the full search strategies for all databases, registers and websites, including any filters and limits used. | Supp. Mat. 4 |
| Selection process | 8 | Specify the methods used to decide whether a study met the inclusion criteria of the review, including how many reviewers screened each record and each report retrieved, whether they worked independently, and if applicable, details of automation tools used in the process. | Methods p 4 |
| Data collection process | 9 | Specify the methods used to collect data from reports, including how many reviewers collected data from each report, whether they worked independently, any processes for obtaining or confirming data from study investigators, and if applicable, details of automation tools used in the process. | Methods p 5-6 |
| Data items | 10a | List and define all outcomes for which data were sought. Specify whether all results that were compatible with each outcome domain in each study were sought (e.g. for all measures, time points, analyses), and if not, the methods used to decide which results to collect. | Methods p 5,  Supp. Mat. 5 |
|  | 10b | List and define all other variables for which data were sought (e.g. participant and intervention characteristics, funding sources). Describe any assumptions made about any missing or unclear information. | Supp. Mat 5 |
| Study risk of bias assessment | 11 | Specify the methods used to assess risk of bias in the included studies, including details of the tool(s) used, how many reviewers assessed each study and whether they worked independently, and if applicable, details of automation tools used in the process. | Methods p 7,  Supp Mat. 7 |
| Effect measures | 12 | Specify for each outcome the effect measure(s) (e.g. risk ratio, mean difference) used in the synthesis or presentation of results. | Table 1 |
| Synthesis methods | 13a | Describe the processes used to decide which studies were eligible for each synthesis (e.g. tabulating the study intervention characteristics and comparing against the planned groups for each synthesis (item #5)). | NA |
|  | 13b | Describe any methods required to prepare the data for presentation or synthesis, such as handling of missing summary statistics, or data conversions. | NA |
|  | 13c | Describe any methods used to tabulate or visually display results of individual studies and syntheses. | NA |
|  | 13d | Describe any methods used to synthesize results and provide a rationale for the choice(s). If meta-analysis was performed, describe the model(s), method(s) to identify the presence and extent of statistical heterogeneity, and software package(s) used. | Methods p 8 |
|  | 13e | Describe any methods used to explore possible causes of heterogeneity among study results (e.g. subgroup analysis, meta-regression). | NA |
|  | 13f | Describe any sensitivity analyses conducted to assess robustness of the synthesized results. | NA |
| Reporting bias assessment | 14 | Describe any methods used to assess risk of bias due to missing results in a synthesis (arising from reporting biases). | Supp. Mat. 7 |
| Certainty assessment | 15 | Describe any methods used to assess certainty (or confidence) in the body of evidence for an outcome. | NA |
| RESULTS | | |  |
| Study selection | 16a | Describe the results of the search and selection process, from the number of records identified in the search to the number of studies included in the review, ideally using a flow diagram. | Figure 1 |
|  | 16b | Cite studies that might appear to meet the inclusion criteria, but which were excluded, and explain why they were excluded. | Supp. Mat 6 |
| Study characteristics | 17 | Cite each included study and present its characteristics. | Table 1 |
| Risk of bias in studies | 18 | Present assessments of risk of bias for each included study. | Supp. Mat. 7 |
| Results of individual studies | 19 | For all outcomes, present, for each study: (a) summary statistics for each group (where appropriate) and (b) an effect estimate and its precision (e.g. confidence/credible interval), ideally using structured tables or plots. | Table 1 |
| Results of syntheses | 20a | For each synthesis, briefly summarise the characteristics and risk of bias among contributing studies. | NA |
|  | 20b | Present results of all statistical syntheses conducted. If meta-analysis was done, present for each the summary estimate and its precision (e.g. confidence/credible interval) and measures of statistical heterogeneity. If comparing groups, describe the direction of the effect. | NA |
|  | 20c | Present results of all investigations of possible causes of heterogeneity among study results. | NA |
|  | 20d | Present results of all sensitivity analyses conducted to assess the robustness of the synthesized results. | NA |
| Reporting biases | 21 | Present assessments of risk of bias due to missing results (arising from reporting biases) for each synthesis assessed. | Supp. Mat. 7 |
| Certainty of evidence | 22 | Present assessments of certainty (or confidence) in the body of evidence for each outcome assessed. | Table 1 |
| DISCUSSION | | |  |
| Discussion | 23a | Provide a general interpretation of the results in the context of other evidence. | Discussion p 3 |
|  | 23b | Discuss any limitations of the evidence included in the review. | Discussion p 5 |
|  | 23c | Discuss any limitations of the review processes used. | Discussion p 2 |
|  | 23d | Discuss implications of the results for practice, policy, and future research. | Discussion p 5 |
| OTHER INFORMATION | | |  |
| Registration and protocol | 24a | Provide registration information for the review, including register name and registration number, or state that the review was not registered. | Methods p 1 |
|  | 24b | Indicate where the review protocol can be accessed, or state that a protocol was not prepared. | Methods p 1 |
|  | 24c | Describe and explain any amendments to information provided at registration or in the protocol. | Supp. Mat 2 |
| Support | 25 | Describe sources of financial or non-financial support for the review, and the role of the funders or sponsors in the review. | Funding |
| Competing interests | 26 | Declare any competing interests of review authors. | Competing interests |
| Availability of data, code and other materials | 27 | Report which of the following are publicly available and where they can be found: template data collection forms; data extracted from included studies; data used for all analyses; analytic code; any other materials used in the review. | Data availability |

*If the information is not provided in the systematic review, give details of where this information is available (e.g., protocol, other published papers (provide citation details), or website (provide the URL)).

Source: Page MJ, McKenzie JE, Bossuyt PM, Boutron I, Hoffmann TC, Mulrow CD, et al. The PRISMA 2020 statement: an updated guideline for reporting systematic reviews. BMJ 2021;372:n71. doi: 10.1136/bmj.n71. This work is licensed under CC BY 4.0. <https://creativecommons.org/licenses/by/4.0/>

# Supplementary material 1b: Synthesis Without Meta-analysis (SWiM) reporting items

| SWiM is intended to complement and be used as an extension to PRISMA | | | |
| --- | --- | --- | --- |
| SWiM reporting item | Item description | Page in manuscript where item is reported | Other* |
| *Methods* | | | |
| 1 Grouping studies for synthesis | 1a) Provide a description of, and rationale for, the groups used in the synthesis (e.g., groupings of populations, interventions, outcomes, study design) | Methods p 8 |  |
|  | 1b) Detail and provide rationale for any changes made subsequent to the protocol in the groups used in the synthesis | Supp. Mat. 2 |  |
| 2 Describe the standardised metric and transformation methods used | Describe the standardised metric for each outcome. Explain why the metric(s) was chosen, and describe any methods used to transform the intervention effects, as reported in the study, to the standardised metric, citing any methodological guidance consulted | Table 1 |  |
| 3 Describe the synthesis methods | Describe and justify the methods used to synthesise the effects for each outcome when it was not possible to undertake a meta-analysis of effect estimates | Methods p 8 |  |
| 4 Criteria used to prioritise results for summary and synthesis | Where applicable, provide the criteria used, with supporting justification, to select the particular studies, or a particular study, for the main synthesis or to draw conclusions from the synthesis (e.g., based on study design, risk of bias assessments, directness in relation to the review question) | NA |  |
| 5 Investigation of heterogeneity in reported effects | State the method(s) used to examine heterogeneity in reported effects when it was not possible to undertake a meta-analysis of effect estimates and its extensions to investigate heterogeneity | Table 1 |  |
| 6 Certainty of evidence | Describe the methods used to assess certainty of the synthesis findings | Table 1 and Supp Mat. 7 |  |
| 7 Data presentation methods | Describe the graphical and tabular methods used to present the effects (e.g., tables, forest plots, harvest plots).  Specify key study characteristics (e.g., study design, risk of bias) used to order the studies, in the text and any tables or graphs, clearly referencing the studies included | Table 1 |  |
| *Results* | | | |
| 8 Reporting results | For each comparison and outcome, provide a description of the synthesised findings, and the certainty of the findings. Describe the result in language that is consistent with the question the synthesis addresses, and indicate which studies contribute to the synthesis | Results p 6-7 |  |
| *Discussion* |  |  |  |
| 9 Limitations of the synthesis | Report the limitations of the synthesis methods used and/or the groupings used in the synthesis, and how these affect the conclusions that can be drawn in relation to the original review question | Discussion p 5 |  |

Source: Campbell M, McKenzie JE, Sowden A, Katikireddi SV, Brennan SE, Ellis S, Hartmann-Boyce J, Ryan R, Shepperd S, Thomas J, Welch V, Thomson H. Synthesis without meta-analysis (SWiM) in systematic reviews: reporting guideline BMJ 2020;368:l6890 <http://dx.doi.org/10.1136/bmj.l6890>

# Supplementary material 2: Differences between the published protocol and manuscript

| **Protocol** | **Difference** | **Reason** |
| --- | --- | --- |
| Screening process: We will use the liberal screening approach to accelerate our screening process. | All studies were screened independently by two reviewers and then later disagreements were resolved. | We used the Covidence systematic review tool for data screening and extraction. Since Covidence does not accommodate a liberal screening approach, in which one reviewer screens and another verifies, all studies had to be screened independently by two reviewers, with any disagreements resolved afterwards. |
| Definition of affective/polarization polarization not included as one of the inclusion criteria. | To be included, political and affective polarization should be defined according to political scientists’ definitions. | We found that definitions of political and affective polarization vary across disciplines. In particular, some fields equate partisanship with polarization, which is not the focus of our review. Therefore, we excluded any studies that used definitions of political and affective polarization that fell outside the political science literature. |
| Studies that measure polarization via social media not considered as one of the exclusion criteria. | Studies that only includes social media data excluded from the systematic review. | We excluded studies based on social media data because their measures of polarization often vary considerably depending on the specific analytic methods used. Moreover, it is difficult to directly compare social media–based indicators (such as text analyses of Twitter posts) with survey-based measures that rely on individual responses. |
| Exposure: Affective and political polarization measured quantitively Outcome: COVID-19 infection risk, COVID-19 hospitalization risk, COVID-19 mortality risk, COVID-19 vaccine uptake, compliance with mask wearing advice, compliance with physical distancing advice, perceived COVID-19 risk. | Cross-sectional studies, includes polarization as the outcome and COVID-19 health outcomes as the exposure are included in the review | In cross-sectional studies where polarization was specified as the outcome, both polarization and COVID-19 health outcomes (as the exposure) were measured at the same time. Because the exposure and outcome are assessed simultaneously, it is not possible to determine the direction of the association, that is, whether polarization leads to COVID-19 health outcomes or vice versa. Based on this, we revised our protocol to clarify that cross-sectional studies assessing both variables concurrently can be included in the review. |
|  |  |  |

# Supplementary material 3: Search strategy

**Review questions**

Question 1: What is the association between political polarization/affective polarization and COVID-19 health outcomes?

Question 2: What is the association between political polarization/affective polarization and COVID-19 related health behaviors?

**Eligibility criteria**

Study selection process: population (P): individuals of any ages and genders; exposure (E): affective and political polarization; comparison (C): not applicable; outcome (O): COVID-19 infection risk, COVID-19 hospitalization risk, COVID-19 mortality risk, COVID-19 vaccine uptake, compliance with mask wearing advice, compliance with physical distancing advice, perceived COVID-19 risk; time (T): studies published from 2019 to 2024; study type (S): studies/manuscripts reporting primary data, irrespective of publication status. No language restriction. Eligible study designs: cohort studies, case-control studies, cross-sectional studies, ecological studies. The exclusion criteria were reviews, editorials or commentaries not reporting original data.

**Sources of information and search strategy**

A systematic literature search was conducted in 12 electronic databases with the help of an experienced medical information specialist: Embase.com, Medline ALL Ovid, Cochrane CENTRAL, Cochrane Covid-19 study register, Web of Science Core Collection, CINAHL, Global Health, PsycInfo, EconLit, WHO Covid-19 research database, iSearch Covid-19 portfolio, and Google Scholar. The search was originally carried out in September 2023. It was updated on 27 November 2024 by re-running all search strategies without date limits. Duplicate records were removed using Deduklick (Borissov et al. 2022). The full search strategy of all databases with results is presented in the Appendix.

The reference lists of the relevant articles and systematic reviews were manually screened. Citationchaser (Haddaway N et al 2021) was used for forward citation tracking of included studies. Experts in the field were consulted for additional publications.

Borissov N, Haas Q, Minder B, Kopp-Heim D, von Gernler M, Janka H, Teodoro D, Amini P. Reducing systematic review burden using Deduklick: A novel, automated, reliable, and explainable deduplication algorithm to foster medical research. Syst. Rev. 2022, 11, 172.

Haddaway NR, Grainger MJ, Gray CT (2021) citationchaser: An R package and Shiny app for forward and backward citations chasing in academic searching. doi: 10.5281/zenodo.4543513

| **Databases names, search dates, results before and after duplications removed.** | | | | |
| --- | --- | --- | --- | --- |
|  | Date search: 8 September 2023 | | Date update: 27 November 2024 | |
|  | Before deduplication | After deduplication^1^ | Before deduplication | After deduplication^2^ |
| Embase.com | 139 | 10 | 184 | 7 |
| Medline ALL Ovid | 222 | 221 | 279 | 48 |
| Cochrane CENTRAL | 5 | 1 | 5 | 0 |
| Cochrane Covid-19 Study Register | 72 | 1 | 79 | 0 |
| Web of Science Core Collection | 591 | 361 | 824 | 153 |
| CINAHL (EBSCOhost) | 62 | 16 | 85 | 5 |
| Global Health (Ovid) | 64 | 14 | 84 | 1 |
| PsycInfo (Ovid) | 119 | 54 | 190 | 29 |
| EconLit (EBSCOhost) | 62 | 23 | 68 | 3 |
| WHO COVID-19 Research Database  (archived in Jan 2024,  no longer searchable) | 797 | 304 (309 initially added to Covidence and 5 removed by Covidence duplication tool) | -- | 0 |
| iSearch COVID-19 Portfolio (NIH) | 397 | 255 | 631 | 115 |
| Google Scholar (first 200) | 200 | 89 (93 initially added to Covidence and 4 removed by Covidence duplication tool) | 100 | 43 |
| Citationchaser (forward citation chasing of 5 refs) | 0 | 0 | 345 | 268 |
| Total | 2730 | 1349 | 2874 | 1655 (672 new hits) |
| Final study count | 2021 (672 new hits were found by the update search following (Bramer W et al 2017)) | | | |
| ^1^1381 duplicate records removed using Deduklick (Borissov N et al 2022)  ^2^1219 duplicate records removed using Deduklick (Borissov N et al 2022) | | | | |

Bramer W, Bain P. Updating search strategies for systematic reviews using EndNote. J Med Libr Assoc. 2017 Jul;105(3):285-289. doi: 10.5195/jmla.2017.183. Epub 2017 Jul 1. PMID: 28670219; PMCID: PMC5490709.

Borissov, N.; Haas, Q.; Minder, B.; Kopp-Heim, D.; von Gernler, M.; Janka, H.; Teodoro, D.; Amini, P. Reducing systematic review burden using Deduklick: A novel, automated, reliable, and explainable deduplication algorithm to foster medical research. Syst. Rev. 2022, 11, 172. <https://doi.org/10.1186/s13643-022-02045-9>

| **Search strategy used in the last update** |
| --- |
| **Embase.com**  ('coronavirus disease 2019'/exp OR 'severe acute respiratory syndrome coronavirus 2'/exp OR 'SARS-related coronavirus'/exp OR 'coronaviridae'/de OR 'coronavirinae'/exp OR 'coronavirus infection'/exp OR (coronavir* OR 'corona virus*' OR covid OR covid19 OR nCoV* OR '2019nCoV' OR 'Severe Acute Respiratory Syndrome*' OR 'SARS-CoV*' OR SARSCoV* OR "corona cris*" OR pandemi*):ab,ti,kw) AND [2019-3000]/py AND ((politic* OR affectiv* OR partisan*) AND (polari*)):ti,ab,kw |
| **Medline ALL (via Ovid)**  (exp Coronavirus/ or exp Coronavirus Infections/ or (coronavir* or corona virus* or covid or covid19 or covid*2 or HCoV* or ncov* or cov2 or cov-2 or 2019nCoV or "novel CoV" or sars-cov* or sarscov* or sars-coronavirus* or Severe Acute Respiratory Syndrome Coronavirus* or "corona cris*" or pandemi*).mp.) and ((20191* or 202*).dp. or 20190101:20301231.(ep).) AND ((politic* OR affectiv* OR partisan*) AND (polari*)).ab,ti,kf |
| **Cochrane Library (via Wiley)**  ((coronavir* OR (corona NEXT virus*) OR covid OR covid19 OR nCoV* OR "2019nCoV" OR "Severe Acute Respiratory Syndrome" OR (SARS NEXT CoV*) OR SARSCoV* OR (corona NEXT cris*) OR pandemi*):ab,ti,kw) AND ((politic* OR affectiv* OR partisan*) AND (polari*)):ti,ab,kw |
| **Cochrane COVID-19 Study Register** <https://covid-19.cochrane.org/>  ((politic* OR affectiv* OR partisan*) AND (polari*)) |
| ***Web of Science Core Collection** (via Clarivate)  TS=(coronavir* OR "corona virus*" OR HCoV* OR nCoV* OR 2019nCoV OR "2019-novel CoV" OR covid OR "covid-19" OR covid19 OR "sars-cov*" or sarscov* or "SARS-coronavirus*" OR "Severe Acute Respiratory Syndrome Coronavirus*" OR "coronavirus cris*" OR "corona cris*" OR pandemi*) AND TS=(((politic* OR affective OR partisan*) AND polari*)) AND PY=(2019-2025)  *Science Citation Index Expanded (1900-present); Social Sciences Citation Index (1900-present) ; Arts & Humanities Citation Index (1975-present) ; Conference Proceedings Citation Index- Science (1990-present) ; Conference Proceedings Citation Index- Social Science & Humanities (1990-present) ; Emerging Sources Citation Index (2018-present) |
| **CINAHL: Cumulative Index to Nursing and Allied Health Literature** with Full Text (via EBSCOhost)  (MH "Coronavirus+" OR MH "Coronavirus Infections+" OR (coronavir* OR corona virus* OR covid OR covid19 OR HCoV* OR nCov* OR cov2 OR cov-2 OR 2019nCoV OR "novel CoV" OR sars-cov* OR sarscov* or sars-coronavirus* OR "Severe Acute Respiratory Syndrome Coronavirus*" OR corona cris* or pandemi*)) AND ((politic* OR affectiv* OR partisan*) AND (polari*))  Published Date: 20190101-20231231 ; Search modes - Boolean/Phrase - Proximity |
| **Global Health** (via Ovid)  (coronavirus disease 2019/ OR severe acute respiratory syndrome coronavirus 2/ OR (coronavir* or corona virus* or covid or covid-19 or covid19 or HCoV* or nCoV* or cov2 or cov-2 or 2019nCoV or "novel CoV" or sars-cov* or sarscov* or sars-coronavirus* or Severe Acute Respiratory Syndrome Coronavirus* or "corona cris*" or pandemi*).mp.) AND ((politic* OR affectiv* OR partisan*) AND (polari*)).ab,ti,id,hw  No date limit necessary |
| **APA PsycINFO** (via OvidSP)  (exp Coronavirus/ or COVID-19/ or SARS-CoV-2/ or (coronavir* or corona virus* or covid or covid-19 or covid19 or HCoV* or nCoV* or cov2 or cov-2 or 2019nCoV or "novel CoV" or sars-cov* or sarscov* or sars-coronavir* or Severe Acute Respiratory Syndrome Coronavirus* or "corona cris*" or pandemic*).mp.) AND ((politic* OR affectiv* OR partisan*) AND (polari*)).ab,ti,id,hw  limit 1 to yr="2019 -Current" |
| **EconLit** (via EBSCOhost)  ((coronavir* or corona virus* or covid or covid-19 or covid19 or HCoV* or nCoV* or cov2 or cov-2 or 2019nCoV or "novel CoV" or sars-cov* or sarscov* or sars-coronavir* or Severe Acute Respiratory Syndrome Coronavirus* or "corona cris*" or pandemic*) AND ((politic* OR affectiv* OR partisan*) AND (polari*)))  Search modes - Boolean/Phrase - Proximity |
| ***WHO COVID-19 Research Database** (via <https://www.who.int/emergencies/diseases/novel-coronavirus-2019/global-research-on-novel-coronavirus-2019-ncov> )  2023: Search in: Title, abstract, subjects via <https://search.bvsalud.org/global-literature-on-novel-coronavirus-2019-ncov/?lang=en>  (politic* OR affectiv* OR partisan*) AND (polari*) *The database was built by BIREME, the Specialized Center of PAHO/AMRO. Its content spanned the time period March 2020 to June 2023. It has now been archived, and no longer searchable since January 2024. |
| **iSearch COVID-19 Portfolio** (NIH) <https://icite.od.nih.gov/covid19/search/> \| unified results from a) OR b)   1. (politic* OR affectiv* OR partisan*) AND (polari*) \|search in title, abstract 2. "political polarization" OR "political polarisation" OR "affective polarization" OR "affective polarisation" \|search in all fields (including the full text) |
| **Google Scholar** (first 200 most relevant results out of 18’000, since 2019)  Political OR affective OR partisan polarization OR polarisation covid OR corona OR sars OR pandemic "health outcome OR outcomes OR behaviour OR behavior" OR hospital OR vaccination OR mortality OR death OR infection study OR trial Update search: (first 100 most relevant results out of 16’500 - time range: 2023-2024) |
| **Citationchaser**  Article input: 35600256, 35779268, 35077546, 33230283, 36595138 (PMIDs of 5 included studies) Searching for all citing articles in Lens.org (forward citation chasing) |

# Supplementary material 4: Eligibility criteria

| **Criterion** | **Description** |
| --- | --- |
| Inclusion criteria | Study population: individuals of any age and gender.  Exposure: Affective and political polarization measured quantitively  Outcome: COVID-19 infection risk, COVID-19 hospitalization risk, COVID-19 mortality risk, COVID-19 vaccine uptake, compliance with mask wearing advice, compliance with physical distancing advice, perceived COVID-19 risk.  Publication type: Manuscript reporting primary data, irrespective of publication status. No language restriction. |
| Eligible study designs | Cohort studies  Case-control studies  Cross-sectional studies  Ecological studies |
| Exclusion criteria | Excluded studies that defined political or affective polarization in ways that did not align with established political science definitions.  Excluded studies that relied solely on social media data, as their polarization measures are not directly comparable to survey-based indicators. |
| Excluded study designs | Reviews, editorials or commentaries not reporting original data |

# Supplementary material 5: Data extraction form

**General Information:**

1) Aim/Objective of the study (Free text)

2) Which study design is used?

A) Cross-sectional study

B) Case-control study

C) Cohort study

D) Survey experiment

E) Other (Free text)

3) Data collection period for polarization/affective polarization (Free text)

4) Which country is the study based? (Drop-down menu)

5) Which questions are addressed in this study?

A) What is the relationship between affective polarization and COVID-19-related outcomes?

B) What is the relationship between political polarization and COVID-19-related outcomes?

6) Comments (Free text)

**Question 1: What is the relationship between affective polarization and COVID-19-related outcomes?**

1) Describe the data collection methods for affective polarization (e.g., online survey, face to face survey) (Free text)

2) Describe the polarized groups that were studied. (e.g., anti-masker vs pro-masker) (Free text)

3) How affective polarization is measured?

A) Feeling thermometer

B) Social distance measure

C) Trust question

D) Character trait measure

E) Other (Free text)

4) Is the affective polarization measure continuous or categorical?

A) Continuous

B) Categorical

5) Which COVID-19 outcomes are assessed? (Multiple choice)

A) COVID-19 infection risk/rate

B) Hospitalization risk/rate

C) Mortality risk/rate

D) COVID-19 vaccine uptake

E) Compliance with mask-wearing advice

F) Compliance with physical distancing advice

G) Perceived COVID-19 risk

H) Level of agreement with the measurements.

I) Other (free text)

6) Describe the data collection methods for the outcome (Statistical office Survey) (Free text)

7) Data collection type (e.g., Aggregated level data, individual-level data) (Free text)

8) What is the measure of effect? (e.g., Odds ratio, Risk ratio) (Free text)

9) What is the method of analysis?

A) Univariable only

B) Univariable and multivariable (if multivariable what was the model adjusted for?) (Free text)

10) Total number of participants? (Free text)

11) Participants' age (mean/median and standard deviation) (Free text)

12) Participants' gender (Free text)

13) Participants' socioeconomic status (Free text)

14) Report the results (Free text)

**Question 2: What is the relationship between political polarization and COVID-19-related outcomes?**

1) Describe the data collection methods for political polarization (e.g., online survey, face to face survey) (Free text)

2) Describe the polarized groups that were studied. (e.g., Republican vs Democrat) (Free text)

3) How political polarization is measured?

A) Voting behavior analysis

B) Network Analysis

C) Text analysis and sentiment analysis

D) Surveys and polls

E) Other (Free text)

4) Is the political polarization measure continuous or categorical?

A) Continuous

B) Categorical

5) Which COVID-19 outcomes are assessed? (Multiple choice)

A) COVID-19 infection risk/rate

B) Hospitalization risk/rate

C) Mortality risk/rate

D) COVID-19 vaccine uptake

E) Compliance with mask-wearing advice

F) Compliance with physical distancing advice

G) Perceived COVID-19 risk

H) Level of agreement with the measurements.

I) Other (Free text)

6) Describe the data collection methods for the outcome (e.g., Statistical office, survey) (Free text)

7) Data collection type (e.g., Aggregated level data, individual-level data) (Free text)

8) What is the measure of effect? (e.g., Odds ratio, Risk ratio) (Free text)

9) What is the method of analysis?

A) Univariable only

B) Univariable and multivariable (if multivariable what was the model adjusted for?) (Free text)

10) Total number of participants? (Free text)

11) Participants' age (mean/median and standard deviation) (Free text)

12) Participants' gender (Free text)

13) Participants' socioeconomic status (Free text)

14) Report the findings (Free text)

# Supplementary material 6: Studies that were excluded at full-text reading and reasons

| Title | Study | DOI / Access links | Notes |
| --- | --- | --- | --- |
| Influence of conspiracy mindset, trust in science, and political affiliation on COVID-19 reactance | Rinato 2023 | https://scholarworks.waldenu.edu/dissertations/11827/ | Exclusion reason: Polarization not assesed; |
| Polarized social distancing: Residents of Republican-majority counties spend more time away from home during the COVID-19 crisis | Roberts 2021 | <https://dx.doi.org/10.1111/ssqu.13101> | Exclusion reason: Polarization not assesed; |
| Dual pandemics of covid-19 and systemic racism: The roles of perceptions of inequities, civic values, and conservatism in mask-wearing behavior | Wegemer 2022 | <https://dx.doi.org/10.1111/asap.12293> | Exclusion reason: Polarization not assesed; |
| Associations Between Governor Political Affiliation and COVID-19 Cases, Deaths, and Testing in the U.S | Neelon 2021 | 10.1016/j.amepre.2021.01.034 | Exclusion reason: Polarization not assesed; |
| Excess mortality versus COVID-19 death rates: a spatial analysis of socioeconomic disparities and political allegiance across US states | Aron 2021 | https://doi.org/10.1111/roiw.12570 | Exclusion reason: Polarization not assesed; |
| Partisan Polarization and Resistance to Elite Messages: Results from Survey Experiments on Social Distancing | Bhanot 2020 | <https://doi.org/10.30636/jbpa.32.178> | Exclusion reason: Polarization not assesed; |
| Political polarization, social fragmentation, and cooperation during a pandemic | Cornelson 2020 | https://www.economics.utoronto.ca/public/workingPapers/tecipa-663.pdf | Exclusion reason: Polarization assessed through partisanship |
| Unmasking Partisanship: Polarization Undermines Public Response to Collective Risk | Milosh 2020 | https://doi.org/10.1016/j.jpubeco.2021.104538 | Exclusion reason: Polarization not assesed |
| Essays on Belief Formation and Political Polarization | Thaler 2020 | https://dash.harvard.edu/entities/publication/3d84ded0-67a9-4389-af1b-05a9703c4c45 | Exclusion reason: Polarization not assesed; |
| The effect of altruism on COVID-19 vaccination rates | Hierro 2022 | 10.1101/2022.06.15.22276430 | Exclusion reason: Other: Preprint of the published paper #560 |
| Associations between COVID-19 vaccine uptake, race/ethnicity, and political party affiliation | Andersen 2023 | 10.1007/s10865-022-00379-2 | Exclusion reason: Polarization not assesed; |
| The Health Costs of Political Identity: Evidence from the US during the COVID-19 Pandemic | Chopra 2022 | https://doi.org/10.1080/21565503.2023.2293118 | Exclusion reason: Polarization assessed through partisanship; |
| The political impact of affective polarization: how partisan animus shapes COVID-19 attitudes | Druckman 2020 | https://www.ipr.northwestern.edu/documents/working-papers/2020/wp-20-35.pdf | Exclusion reason: Other: No health outcome; |
| The effect of political polarization on social distance stances in the Brazilian COVID-19 scenario | Ebeling 2021 | https://journals-sol.sbc.org.br/index.php/jidm/article/download/1889/1776 | Exclusion reason: Other: Twitter data exclude; |
| Analysis of the influence of political polarization in the vaccination stance: the Brazilian COVID-19 scenario | Ebeling 2022 | https://doi.org/10.1609/icwsm.v16i1.19281 | Exclusion reason: Other: Twitter data exclude; |
| The harmful effects of partisan polarization on health | Fraser 2022 | https://doi.org/10.1093/pnasnexus/pgac011 | Exclusion reason: Not COVID-19 related outcome; |
| Partisanship, health behavior, and policy attitudes in the early stages of the COVID-19 pandemic | Gadarian 2021 | https://doi.org/10.1371/journal.pone.0249596 | Exclusion reason: Polarization not assesed; |
| Death by political party: The relationship between COVID‐19 deaths and political party affiliation in the United States | Gao 2021 | 10.1002/wmh3.435 | Exclusion reason: Polarization assessed through partisanship; |
| Partisan differences in physical distancing are linked to health outcomes during the COVID-19 pandemic | Gollwitzer 2020 | https://doi.org/10.1038/s41562-020-00977-7 | Exclusion reason: Polarization not assesed; |
| Partisanship, messaging, and the COVID-19 vaccine: evidence from survey experiments | Golos 2022 | 10.1177/08901171211049241 | Exclusion reason: Polarization not assesed; |
| Understanding the influence of political orientation, social network, and economic recovery on COVID-19 vaccine uptake among Americans | Hao 2022 | https://doi.org/10.1016/j.vaccine.2022.02.066 | [Exclusion reason: Polarization not assessed quantitatively;](https://pubmed.ncbi.nlm.nih.gov/35227522/) |
| The association between vaccination status identification and societal polarization | Henkel 2023 |  | [Exclusion reason: Polarization not assesed;](https://doi.org/10.1038/s41562-022-01469-6) |
| Ideology and COVID-19 Vaccination Intention: Perceptual Mediators and Communication Moderators | Jiang 2022 | 10.1080/10810730.2022.2117438 | Exclusion reason: Polarization not assesed; |
| Trump vs. the GOP: Political Determinants of COVID-19 Vaccination | Jung 2023 | 10.1007/s11109-023-09882-x | Exclusion reason: Polarization not assesed; |
| Social Norms, Political Polarization, and Vaccination Attitudes: Evidence from a Survey Experiment in Turkey | Kaba 2023 | https://doi.org/10.1016/j.euroecorev.2024.104818 | Exclusion reason: Not COVID-19 related outcome; |
| Partisanship and Covid-19 vaccination in the UK | Klymak 2022 | https://doi.org/10.1038/s41598-022-23035-w | Exclusion reason: Polarization not assesed; |
| Factors influencing Covid-19 vaccine acceptance across subgroups in the United States: Evidence from a conjoint experiment | Kreps 2021 | https://doi.org/10.1016/j.vaccine.2021.04.044 | Exclusion reason: Polarization not assesed; |
| The Impact of Political Polarization on the COVID-19 Vaccine Hesitancy in the United States: A Qualitative Study | Ma 2023 | https://doi.org/10.5539/jpl.v16n2p37 | Exclusion reason: Polarization not assessed quantitatively; |
| The real cost of political polarization: Evidence from the COVID-19 pandemic | Makridis 2020 | https://www.econbiz.de/Record/the-real-cost-of-political-polarization-evidence-from-the-covid-19-pandemic-makridis-christos/10012311202 | Exclusion reason: Polarization not assesed; |
| Identity versus fear of death: political polarization under the COVID-19 pandemic in Brazil | Medeiros 2021 | 10.1007/978-3-030-77602-2_10 | Exclusion reason: Polarization not assesed; |
| Bridging the divide: does social capital moderate the impact of polarization on health? | Panagopoulos 2022 | 10.1177/10659129211034561 | Exclusion reason: conducted prior to COVID pandemic; |
| Effects of living conditions, political orientation, and empathy on behaviors and attitudes during the COVID-19 pandemic: a study in the Brazilian context | Sampaio 2023 | 10.1007/s43076-021-00130-x | Exclusion reason: Polarization assessed through partisanship; |
| … -19 Mortality And The County-Level Partisan Divide In The United States: Study examines the association between COVID-19 mortality and county-level political party … | Sehgal 2022 | 10.1377/hlthaff.2022.00085 | Exclusion reason: Polarization not assesed; Maximilian Filsinger (2024-03-21 21:08:10)(Select): Partisanship |
| Affective partisan polarization and moral dilemmas during the COVID-19 pandemic | Stoetzer 2023 | doi:10.1017/psrm.2022.13 | Exclusion reason: Wrong study design; |
| Drivers of COVID-19 booster uptake among nurses | Viskupič 2023 | https://doi.org/10.1016/j.ajic.2022.11.014 | [Exclusion reason: Polarization not assesed;](https://www.ncbi.nlm.nih.gov/pmc/articles/PMC9683517/) |
| Divided by the Jab: On the Nature, Origins, and Consequences of COVID-19 Vaccination Identities | Wagner 2022 | https://doi.org/10.1080/17457289.2024.2352449 | [Exclusion reason: Not COVID-19 related outcome;](https://osf.io/preprints/osf/zcas8) |
| Exploring the relationship between political partisanship and COVID-19 vaccination rate | Ye 2023 | https://doi.org/10.1093/pubmed/fdab364 | Exclusion reason: Polarization assessed through partisanship; |
| Ideology and compliance with health guidelines during the COVID-19 pandemic: A comparative perspective | Becher 2021 | 10.1111/ssqu.13035 | Exclusion reason: Polarization not assesed; |
| Analysis of the influence of political polarization in the vaccination stance: the Brazilian COVID-19 scenario | Ebeling 2021 | https://doi.org/10.1609/icwsm.v16i1.19281 | Exclusion reason: Other: Duplication |
| Predictors of Death Rate during the COVID-19 Pandemic | Feinhandler 2020 | 10.3390/healthcare8030339 | Exclusion reason: Polarization assessed through partisanship; |
| An Early Examination: Psychological, Health, and Economic Correlates and Determinants of Social Distancing Amidst COVID-19 | Im 2021 | 10.3389/fpsyg.2021.589579 | Exclusion reason: Polarization not assesed; |
| A longitudinal study of vaccine hesitancy attitudes and social influence as predictors of COVID-19 vaccine uptake in the US | Latkin 2022 | 10.1080/21645515.2022.2043102 | Exclusion reason: Polarization not assesed; |
| Leveraging 13 million responses to the U.S. COVID-19 Trends and Impact Survey to examine vaccine hesitancy, vaccination, and mask wearing, January 2021-February 2022 | Nguyen 2022 | 10.1186/s12889-022-14286-3 | Exclusion reason: Polarization not assesed; |
| Politics of COVID-19 vaccine mandates: Left/right-wing authoritarianism, social dominance orientation, and libertarianism | Peng 2022 | 10.1016/j.paid.2022.111661 | Exclusion reason: Polarization not assesed; |
| <p>Political Affiliation and Race Associated With Parents' Intentions to Have a COVID-19 Vaccination but Not With History of Refusing to Vaccinate a Child: A Cross-Sectional Study</p> | Roess 2021 | [10.21203/rs.3.rs-469429/v1](http://rs.3.rs/) | Exclusion reason: Polarization not assesed; |
| Using trajectory modeling of spatio-temporal trends to illustrate disparities in COVID-19 death in flint and Genesee County, Michigan | Sadler 2022 | 10.1016/j.sste.2022.100536 | Exclusion reason: Polarization not assesed; |
| Drivers of COVID-19 booster uptake among nurses | Viskupič 2022 | 10.1016/j.ajic.2022.11.014 | Exclusion reason: Polarization assessed through partisanship; |
| Involvement of political and socio-economic factors in the spatial and temporal dynamics of COVID-19 outcomes in Brazil: A population-based study | Xavier 2022 | 10.1016/j.lana.2022.100221 | Exclusion reason: Polarization not assesed; |
| Polarization and public health: Partisan differences in social distancing during the coronavirus pandemic | Allcott 2020 | <https://dx.doi.org/10.1016/j.jpubeco.2020.104254> | Exclusion reason: Polarization not assesed; |
| Association of Republican partisanship with US citizens' mobility during the first period of the COVID crisis | Barbalat 2022 | <https://dx.doi.org/10.1038/s41598-022-12790-5> | Exclusion reason: Polarization not assesed; |
| Risk perceptions and politics: Evidence from the COVID-19 pandemic | Barrios 2021 | <https://dx.doi.org/10.1016/j.jfineco.2021.05.039> | Exclusion reason: Polarization not assesed; |
| Perceived risk, political polarization, and the willingness to follow COVID-19 mitigation guidelines | Block 2022 | <https://dx.doi.org/10.1016/j.socscimed.2022.115091> | Exclusion reason: Polarization assessed through partisanship; |
| Assessing COVID-19 pandemic policies and behaviours and their economic and educational trade-offs across US states from Jan 1, 2020, to July 31, 2022: an observational analysis | Bollyky 2023 | <https://dx.doi.org/10.1016/S0140-6736(23)00461-0> | Exclusion reason: Polarization not assesed; |
| Political polarization in US residents' COVID-19 risk perceptions, policy preferences, and protective behaviors | BruinedeBruin 2020 | <https://dx.doi.org/10.1007/s11166-020-09336-3> | Exclusion reason: Polarization assessed through partisanship; |
| Uncooperative society, uncooperative politics or both? Trust, polarization, populism and COVID-19 deaths across European regions | Charron 2022 | <https://dx.doi.org/10.1111/1475-6765.12529> | Exclusion reason: Other; Duplicate; |
| Relationship between political partisanship and COVID-19 deaths: future implications for public health | Chen 2022 | <https://dx.doi.org/10.1093/pubmed/fdab136> | Exclusion reason: Polarization not assesed; |
| Partisan polarization, historical heritage, and public health: Exploring COVID-19 outcomes | Curtis 2022 | <https://dx.doi.org/10.1002/wmh3.543> | Exclusion reason: Polarization not assesed; |
| Polarized Public Opinion About Public Health During the COVID-19 Pandemic: Political Divides and Future Implications | Findling 2022 | <https://dx.doi.org/10.1001/jamahealthforum.2022.0016> | Exclusion reason: Polarization not assesed; |
| The politicized pandemic: Ideological polarization and the behavioral response to COVID-19 | Grimalda 2023 | <https://dx.doi.org/10.1016/j.euroecorev.2023.104472> | Exclusion reason: Polarization not assessed quantitatively; |
| Personality and Its Partisan Political Correlates Predict U.S. State Differences in Covid-19 Policies and Mask Wearing Percentages | Heyman 2021 | <https://dx.doi.org/10.3389/fpsyg.2021.729774> | Exclusion reason: Polarization not assesed; |
| Social mobilization and polarization can create volatility in COVID-19 pandemic control | Hong 2021 | <https://dx.doi.org/10.1007/s41109-021-00356-9> | Exclusion reason: Not COVID-19 related outcome; |
| Political polarization on COVID-19 pandemic response in the United States | Kerr 2021 | <https://dx.doi.org/10.1016/j.paid.2021.110892> | Exclusion reason: Polarization not assesed; |
| The polarization of politics and public opinion and their effects on racial inequality in COVID mortality | Lo 2022 | <https://dx.doi.org/10.1371/journal.pone.0274580> | Exclusion reason: Polarization assessed through partisanship; |
| Anti-Vaxxers, Politicization of Science, and the Need for Trust in Pandemic Response | May.20 | <https://dx.doi.org/10.1080/10810730.2020.1864519> | Exclusion reason: Not original data; |
| Polarization, partisanship, and pandemic: The relationship between county-level support for Donald Trump and the spread of Covid-19 during the spring and summer of 2020 | Morris 2021 | <https://dx.doi.org/10.1111/ssqu.13053> | Exclusion reason: Polarization not assesed; |
| Political beliefs affect compliance with government mandates | Painter 2021 | <https://dx.doi.org/10.1016/j.jebo.2021.03.019> | Exclusion reason: Polarization not assesed; |
| Beliefs About COVID-19 in Canada, the United Kingdom, and the United States: A Novel Test of Political Polarization and Motivated Reasoning | Pennycook 2022 | <https://dx.doi.org/10.1177/01461672211023652> | Exclusion reason: Polarization not assesed; |
| The association between the proportion of Brexiters and COVID-19 death rates in England | Phalippou 2023 | <https://dx.doi.org/10.1016/j.socscimed.2023.115826> | Exclusion reason: Polarization not assesed; |
| Both trust in, and polarization of trust in, relevant sciences have increased through the COVID-19 pandemic | Radrizzani 2023 | <https://dx.doi.org/10.1371/journal.pone.0278169> | Exclusion reason: Polarization not assesed; |
| Individual-Community Misalignment in Partisan Identity Predicts Distancing From Norms During the COVID-19 Pandemic | Reid 2023 | <https://dx.doi.org/10.1177/19485506221121204> | Exclusion reason: Polarization not assesed; |
| Morbid Polarization: Exposure to COVID-19 and Partisan Disagreement about Pandemic Response | Rodriguez 2022 | <https://dx.doi.org/10.1111/pops.12810> | Exclusion reason: Polarization not assessed quantitatively; |
| Analysing COVID-19 outcomes in the context of the 2019 Global Health Security (GHS) Index | Rose 2021 | <https://dx.doi.org/10.1136/bmjgh-2021-007581> | Exclusion reason: Polarization not assesed; |
| Confidence in political leaders can slant risk perceptions of COVID-19 in a highly polarized environment | Shao 2020 | <https://dx.doi.org/10.1016/j.socscimed.2020.113235> | Exclusion reason: Polarization not assesed; |
| Risk perception, but also political orientation, modulate behavioral response to COVID-19: A randomized survey experiment | Torrente 2022 | <https://dx.doi.org/10.3389/fpsyg.2022.900684> | Exclusion reason: Polarization not assesed; |
| Right-wing ideological constraint and vaccine refusal: The case of the COVID-19 vaccine in Norway | Wollebaek 2022 | <https://dx.doi.org/10.1111/1467-9477.12224> | Exclusion reason: Polarization not assesed; |
| Social asset or social liability? How partisanship moderates the relationship between social capital and Covid-19 vaccination rates across United States counties | Zhang 2022 | <https://dx.doi.org/10.1016/j.socscimed.2022.115325> | Exclusion reason: Polarization assessed through partisanship; |
| Vaccination, politics and COVID-19 impacts | Albrecht 2022 | 10.1186/s12889-021-12432-x | Exclusion reason: Polarization not assesed; |
| Polarization and Accountability in Covid Times | Beramendi 2022 | 10.3389/fpos.2021.728341 | Exclusion reason: Polarization assessed through partisanship; |
| Partisanship Unmasked? The Role of Politics and Social Norms in COVID-19 Mask-Wearing Behavior | Carey 2022 | 10.1017/xps.2022.20 | Exclusion reason: Polarization assessed through partisanship; |
| COVID-19 Vaccine Hesitancy Is the New Terrain for Political Division among Americans | Cowan 2021 | 10.1177/23780231211023657 | Exclusion reason: Polarization assessed through partisanship; |
| How Affective Polarization Shapes Americans' Political Beliefs: A Study of Response to the COVID-19 Pandemic | Druckman 2021 | 10.1017/xps.2020.28 | Exclusion reason: Not COVID-19 related outcome; |
| Partisanship and the Politics of COVID Vaccine Hesitancy | Jones 2022 | 10.1086/719918 | Exclusion reason: Polarization not assesed; |
| Along party Lines: Examining the gubernatorial party difference in COVID-19 mortality rates in US Counties | Lhila 2023 | 10.1016/j.pmedr.2023.102142 | Exclusion reason: Polarization not assesed; |
| Unmasking partisanship: Polarization undermines public response to collective risk | Milosh 2021 | 10.1016/j.jpubeco.2021.104538 | Exclusion reason: Polarization assessed through partisanship; |
| In different worlds: The contributions of polarization and platforms to partisan (mis)perceptions | Overgaard 2023 | 10.1177/14614448231176551 | Exclusion reason: Not COVID-19 related outcome; |
| Politicization of a Pathogen: A Prospective Longitudinal Study of COVID-19 Responses in a Nationally Representative US Sample | Relihan 2023 | 10.1111/pops.12894 | Exclusion reason: Polarization assessed through partisanship; |
| Effects of trust, risk perception, and health behavior on COVID-19 disease burden: Evidence from a multi-state US survey | Ridenhour 2022 | 10.1371/journal.pone.0268302 | Exclusion reason: Polarization not assesed; |
| The effects of partisan framing on COVID-19 attitudes: Experimental evidence from early and late pandemic | Wichowsky 2022 | 10.1177/20531680221096049 | Exclusion reason: Polarization not assessed quantitatively; |
| Associations between governor political affiliation and COVID-19 cases and deaths in the United States (preprint) | Brian 2020 | 10.1101/2020.10.08.20209619 | Exclusion reason: Polarization not assesed; |
| Moral Identity predicts Adherence to COVID-19 Mitigation Procedures depending on Political Ideology: A Comparison between USA and New Zealand (preprint) | Cillian 2022 | [10.31234/osf.io/dmjrs](http://osf.io/dmjrs) | Exclusion reason: Polarization not assesed; |
| Risk perception, but also political orientation, modulate behavioral response to COVID-19: a randomized survey experiment. (preprint) | Fernando 2022 | [10.31234/osf.io/fwmqp](http://osf.io/fwmqp) | Exclusion reason: Polarization not assesed; |
| COVID-19 and vaccine hesitancy: A longitudinal study | Fridman 2021 | 10.1371/journal.pone.0250123 | Exclusion reason: Polarization not assesed; |
| Beliefs about COVID-19 in Canada, the U.K., and the U.S.A.: A novel test of political polarization and motivated reasoning (preprint) | Gordon 2020 | [10.31234/osf.io/zhjkp](http://osf.io/zhjkp) | Exclusion reason: Polarization not assessed quantitatively; |
| The Political Division toward COVID-19, Vaccines, Contact Tracing Apps, and A Future Pandemic Scenario in the United States: A Survey Result from A National Representative Sample (preprint) | Haijing 2023 | 10.1101/2023.07.20.23292950 | Exclusion reason: Polarization not assesed; Aziz Mert Ipekci (2024-03-20 20:51:30)(Select): Partizanship; |
| Polarization and Public Health: Partisan Differences in Social Distancing during COVID-19 (preprint) | Hunt 2021 | 10.2139/ssrn.3570274 | Exclusion reason: Polarization not assesed; |
| The Costs of Polarizing a Pandemic: Antecedents, Consequences, and Lessons (preprint) | JayJosephVan 2022 | [10.31234/osf.io/qdb97](http://osf.io/qdb97) | Exclusion reason: Polarization assessed through partisanship; |
| The harmful effects of denial: When political polarization meets covid-19 social distancing | Leone 2021 | 10.23870/marlas.327 | Exclusion reason: Polarization not assesed; |
| The effect of altruism on COVID-19 vaccination rates (preprint) | Luis 2022 | 10.1101/2022.06.15.22276430 | Exclusion reason: Other; Duplicate |
| Unmasking Partisanship: Polarization Undermines Public Response to Collective Risk (preprint) | Maria 2020 | 10.2139/ssrn.3664779 | Exclusion reason: Polarization not assesed; |
| 'Distancers' and 'non-distancers'? The potential social psychological impact of moralizing COVID-19 mitigating practices on sustained behaviour change | Prosser 2020 | 10.1111/bjso.12399 | Exclusion reason: Not original data; |
| Analysis of the influence of political polarization in the vaccination stance: the Brazilian COVID-19 scenario (preprint) | Régis 2021 | 2110.03382v1 | Exclusion reason: Not COVID-19 related outcome; |
| Partisan Differences in Social Distancing Response to COVID-19 in the United States (preprint) | Zhiqi 2021 | 10.2139/ssrn.3849921 | Exclusion reason: Other; The study was removed from SSRN after author's request; |
| SOCIO-ECONOMIC ASPECTS OF HEALTHCARE POLICY IN THE CONTEXT OF PARTISAN BIAS EFFECT | Bel 2023 | https://shorturl.at/jdhiq | Exclusion reason: Not original data; |
| COVID-19 stigmatization after the development of effective vaccines: Vaccination behavior, attitudes, and news sources | DesJarlais 2023 | 10.1371/journal.pone.0283467 | Exclusion reason: Polarization not assesed; |
| The politics of COVID-19: Differences between U.S. red and blue states in COVID-19 regulations and deaths | DominikGüss 2023 | 10.1016/j.hpopen.2023.100107 | Exclusion reason: Polarization assessed through partisanship; |
| A population level study on the determinants of COVID-19 vaccination rates at the US county level | Dong 2024 | https://doi.org/10.1038/s41598-024-54441-x | Exclusion reason: Polarization not assesed; |
| Political variations in pandemic lifestyles and COVID-19 vaccination by age cohort in the United States | Dowd-Arrow 2023 | https://doi.org/10.1016/j.ypmed.2023.107525 | Exclusion reason: Polarization not assesed; |
| The Mortality of Politics: An American Paradox | Evans 2024 | <https://dx.doi.org/10.1159/000541912> | Exclusion reason: Polarization assessed through partisanship; |
| Asymmetric affective polarization regarding COVID-19 vaccination in six European countries | Filsinger 2024 | <https://dx.doi.org/10.1038/s41598-024-66756-w> | Exclusion reason: Not COVID-19 related outcome; |
| Counterfactual thinking may attenuate polarization of COVID-19 prevention behavior | GarciaFerres 2023 | <https://dx.doi.org/10.1111/spc3.12891> | Exclusion reason: Polarization not assesed; |
| Polarized Perspectives on Health Equity: Results from a Nationally Representative Survey on US Public Perceptions of COVID-19 Disparities in 2023 | Gollust 2024 | <https://dx.doi.org/10.1215/03616878-11066304> | Exclusion reason: Not COVID-19 related outcome; |
| The Political Division toward COVID-19, Vaccines, Contact Tracing Apps, and A Future Pandemic Scenario in the United States: A Survey Result from A National Representative Sample | Hao 2023 | 10.1101/2023.07.20.23292950 | Exclusion reason: Polarization assessed through partisanship; |
| What Factors Affect People's Opinions Toward COVID-19 and Contact Tracing Apps in the United States: A Random Sample Survey | Hao 2024 | 10.4018/ijhisi.353901 | Exclusion reason: Polarization assessed through partisanship; |
| COVID-19 and perceived risk: examining mortality salience and political decision-making during a pandemic | Harvell-Bowman 2024 | 10.1080/13669877.2024.2350710 | Exclusion reason: Polarization not assesed; |
| Social norms, political polarization, and vaccination attitudes: Evidence from a survey experiment in Turkey | Kaba 2024 | 10.1016/j.euroecorev.2024.104818 | Exclusion reason: Not COVID-19 related outcome; |
| The Politicization of the COVID-19 Pandemic | Kirbis 2024 | <https://dx.doi.org/10.1007/978-3-031-61943-4_9> | Exclusion reason: Not original data; |
| Responses to political partisans are shaped by a COVID-19-sensitive disease avoidance psychology: A longitudinal investigation of functional flexibility | Ko 2024 | <https://dx.doi.org/10.1037/amp0001318> | Exclusion reason: Polarization assessed through partisanship; |
| The politics of vaccination: a closer look at the beliefs, social norms, and prevention behaviors related to COVID-19 vaccine uptake within two US political parties | Konstantopoulos 2024 | 10.1080/13548506.2023.2283401 | Exclusion reason: Polarization assessed through partisanship; |
| Examining American attitudes toward vaccination during the COVID-19 pandemic from the perspective of negative and positive rights | Koong 2023 | <https://dx.doi.org/10.1017/pls.2023.17> | Exclusion reason: Polarization not assesed; |
| The Wasserstein Bipolarization Index: A New Measure of Public Opinion Polarization, with an Application to Cross-Country Attitudes toward COVID-19 Vaccination Mandates | Lee 2024 | https://ideas.repec.org/p/osf/osfxxx/etzh3.html | Exclusion reason: Wrong study design; |
| Association Between Risk Perceptions of COVID-19, Political Ideology, and Mask-Wearing Behavior After the Outbreak: A Cross-Sectional Survey in South Korea | Lee 2024 | 10.2147/RMHP.S463739 | Exclusion reason: Polarization not assesed; |
| COVID‐19 Vaccination Acceptance: A Case of Interplay Between Political and Health Dimensions | Lima 2023 | 10.1111/pops.12893 | Exclusion reason: Polarization assessed through partisanship; |
| Local Norms, Political Partisanship, and Pandemic Response: Evidence from the United States | Lipsitz 2024 | 10.1017/s1537592723002864 | Exclusion reason: Polarization assessed through partisanship; |
| State-to-state differences in US COVID-19 outcomes: searching for explanations | Lurie 2023 | 10.1016/S0140-6736(23)00726-2 | Exclusion reason: Not original data; |
| Political party affiliation, social identity cues, and attitudes about protective mask-wearing during the COVID-19 pandemic in Germany | Magnus 2024 | https://doi.org/10.1371/journal.pone.0302399 | Exclusion reason: Polarization assessed through partisanship; |
| The Psychological Determinants of Avoiding Crowded Areas: An International and Political Investigation | Matsunaga 2023 | <https://dx.doi.org/10.5964/jspp.9819> | Exclusion reason: Polarization not assesed; |
| Understanding the interconnection between public health and political behaviors in a politically polarized context: The impact of race, political attitudes, and policy factors on the US COVID-19 pandemic response | Nkouaga 2023 | https://digitalrepository.unm.edu/pols_etds/94/ | Exclusion reason: Polarization assessed through partisanship; |
| How Partisanship and Polarization Affected COVID-19 Policy and Outcomes in the United States | Olsen 2023 | https://etd.library.emory.edu/concern/etds/d791sh418 | Exclusion reason: Polarization assessed through partisanship; |
| <p><strong>Psychosocial Factors Influencing Vaccine Hesitancy: A Study in the Context of COVID-19</strong></p> | Rahel 2024 | [10.21203/rs.3.rs-4156314/v1](http://rs.3.rs/) | Exclusion reason: Polarization not assesed; |
| Affective Polarization Between Opinion-Based Groups in a Context of Low Partisan Discord: Measuring Its Prevalence and Consequences | Schieferdecker 2024 | 10.1093/ijpor/edae009 | Exclusion reason: Not COVID-19 related outcome; |
| Pandemic Politics: Political Worldviews and COVID-19 Beliefs and Practices in an Unsettled Time | Shepherd 2020 | 10.1177/2378023120972575 | Exclusion reason: Polarization assessed through partisanship; |
| COVID-19 Vaccination Experiences in Social Circles Influence Preferences for COVID-19 Vaccine Mandates: An Online Survey of the United States Population | Skidmore 2024 | 10.20944/preprints202401.1470.v1 | Exclusion reason: Polarization not assesed; |
| Acceptance of Political Restrictions During the COVID-19 Pandemic-A Comparative Study of Austria and Hungary, the Manifestation of Political Polarization | Susánszky 2023 | https://doi.org/10.1177/00207152231187196 | Exclusion reason: Wrong study design; |
| The Costs of Polarizing a Pandemic: Antecedents, Consequences, and Lessons | VanBavel 2024 | <https://dx.doi.org/10.1177/17456916231190395> | Exclusion reason: Polarization not assesed; |
| Excess death rates for republican and democratic registered voters in Florida and Ohio during the COVID-19 pandemic | Wallace 2023 | https://doi.org/10.1001/jamainternmed.2023.1154 | Exclusion reason: Polarization assessed through partisanship; |
| A test of vaccine endorsement by political in‐ versus out‐group sources: Effect on vaccination likelihood and exploration of mediation through perceived bias and liking | Wallace 2023 | 10.1111/spc3.12818 | Exclusion reason: Polarization not assesed; |
| Vaccine Attitude Spillover: COVID-19 Vaccination, Flu Vaccination, and Political Polarization | Walsh 2024 | https://ecommons.udayton.edu/stander_posters/3632/ | Exclusion reason: Wrong study design; |
| Age and partisan self-identification predict uptake of additional COVID-19 booster doses: Evidence from a longitudinal study | Wiltse 2023 | https://doi.org/10.1016/j.pmedr.2023.102407 | Exclusion reason: Polarization assessed through partisanship; |
| Regional and Temporal Patterns of Partisan Polarization during the COVID-19 Pandemic in the United States and Canada | Yang 2024 | https://doi.org/10.48550/arXiv.2407.02807 | Exclusion reason: Polarization assessed through partisanship; |

# Supplementary material 7: Risk of bias assessment

| **Checklist for analytical cross-sectional studies - Joanna Briggs Institute.** | | | | | | | | |
| --- | --- | --- | --- | --- | --- | --- | --- | --- |
| Study ID | Were the criteria for inclusion in the sample clearly defined? | Were the study subjects and the setting described in detailed? | Was the exposure measured in a valid and reliable way? | Were objective, standard criteria used for measurement of the condition? | Were confounding factors identified? | Were strategies to deal with confounding factors adequate to control confounding? | Were the outcomes measured in a valid and reliable way? | Was appropriate statistical analysis used? |
| [Charron et al., 2023](#_ENREF_6) | Unclear | No | Yes | Yes | Yes | Yes | Yes | Yes |
| [Cornelson & Miloucheva, 2022](#_ENREF_7) | Yes | Yes | Yes | Unclear | Yes | Yes | Yes | Yes |
| [Dolman et al., 2023](#_ENREF_9) | Unclear | No | Yes | Yes | Yes | Yes | Yes | Yes |
| [J. N. Druckman et al., 2021](#_ENREF_11) | Yes | Yes | Yes | Yes | Yes | Yes | Yes | Yes |
| [Hierro et al., 2023](#_ENREF_20) ^1^ | Yes | Unclear | Unclear | Yes | Yes | Yes | Yes | Yes |
| [Kim & Pelc, 2024](#_ENREF_26) ^1^ | Unclear | Yes | Yes | Yes | Yes | Yes | Yes | Yes |
| [Nezi, 2024](#_ENREF_33) | Unclear | No | Yes | Yes | Yes | No | Yes | Yes |
| [Wagner & Eberl, 2024](#_ENREF_39) | Yes | Yes | Yes | Yes | Yes | Yes | Yes | Yes |
| ([Wróblewski & Meler, 2024](#_ENREF_40)) | Yes | No | Yes | Yes | No | No | Yes | No |

Source: Moola S, Munn Z, Tufanaru C, Aromataris E, Sears K, Sfetcu R, Currie M, Qureshi R, Mattis P, Lisy K, Mu P-F. Chapter 7: Systematic reviews of etiology and risk . In: Aromataris E, Munn Z (Editors)*. JBI Manual for Evidence Synthesis.*JBI, 2020. Available from <https://synthesismanual.jbi.global>

| **Risk of bias questions** | | | | | |
| --- | --- | --- | --- | --- | --- |
| Study ID | Could the inclusion criteria or the sampling strategy lead to bias? (Selection bias) | Is there a difference between responders and non-responders? | Could the difference between responders and non-responders lead to bias? | Could the measurement of the exposure lead to bias? | Could measurement of the outcome lead to bias? |
| [Charron et al., 2023](#_ENREF_6) | Unclear | Unclear | Unclear | Low risk of bias | Low risk of bias |
| [Cornelson & Miloucheva, 2022](#_ENREF_7) | High risk of bias | Unclear | Unclear | Low risk of bias | Low risk of bias |
| [Dolman et al., 2023](#_ENREF_9) | Unclear | Unclear | Unclear | Low risk of bias | Low risk of bias |
| [J. N. Druckman et al., 2021](#_ENREF_11) | High risk of bias | Unclear | Unclear | Low risk of bias | Low risk of bias |
| [Hierro et al., 2023](#_ENREF_20) ^1^ | Low risk of bias | Unclear | Unclear | Unclear | Low risk of bias |
| [Kim & Pelc, 2024](#_ENREF_26) ^1^ | High risk of bias | Unclear | Unclear | Low risk of bias | Low risk of bias |
| [Nezi, 2024](#_ENREF_33) | Unclear | Unclear | Unclear | Low risk of bias | Low risk of bias |
| [Wagner & Eberl, 2024](#_ENREF_39) | Low risk of bias | Unclear | Unclear | Low risk of bias | Low risk of bias |
| [Wróblewski & Meler, 2024](#_ENREF_40) | Low risk of bias | Low risk of bias | Unclear | Unclear | Low risk of bias |

Source: Tonia T, Buitrago-Garcia D, Peter NL, Mesa-Vieira C, Li T, Furukawa TA, et al. BMJ Ment Health 2023 Vol. 26 Issue 1 DOI: 10.1136/bmjment-2023-300694
